# Electron Correlation or Basis Set Quality: How to Obtain Converged and Accurate NMR Shieldings for the Third-Row Elements?

**DOI:** 10.3390/molecules27238230

**Published:** 2022-11-25

**Authors:** Kacper Rzepiela, Jakub Kaminský, Aneta Buczek, Małgorzata A. Broda, Teobald Kupka

**Affiliations:** 1 Faculty of Chemistry, University of Opole, 48 Oleska Street, 46-052 Opole, Poland; 2 Institute of Organic Chemistry and Biochemistry of the CAS, Flemingovo nám. 2, 166 10 Prague, Czech Republic

**Keywords:** NMR shieldings, basis set dependence, third-row elements

## Abstract

The quality of theoretical NMR shieldings calculated at the quantum-chemical level depends on various theoretical aspects, of which the basis set type and size are among the most important factors. Nevertheless, not much information is available on the basis set effect on theoretical shieldings of the NMR-active nuclei of the third row. Here, we report on the importance of proper basis set selection to obtain accurate and reliable NMR shielding parameters for nuclei from the third row of the periodic table. All calculations were performed on a set of eleven compounds containing the elements Na, Mg, Al, Si, P, S, or Cl. NMR shielding tensors were calculated using the SCF-HF, DFT-B3LYP, and CCSD(T) methods, combined with the Dunning valence aug-cc-pV*X*Z, core-valence aug-cc-pCV*X*Z, Jensen polarized-convergent aug-pcSseg-*n* and Karlsruhe x2c-Def2 basis set families. We also estimated the complete basis set limit (CBS) values of the NMR parameters. Widely scattered nuclear shieldings were observed for the Dunning polarized-valence basis set, which provides irregular convergence. We show that the use of Dunning core-valence or Jensen basis sets effectively reduces the scatter of theoretical NMR results and leads to their exponential-like convergence to CBS. We also assessed the effect of vibrational, temperature, and relativistic corrections on the predicted shieldings. For systems with single bonds, all corrections are relatively small, amounting to less than 4% of the CCSD(T)/CBS value. Vibrational and temperature corrections were less reliable for H_3_PO and HSiCH due to the high anharmonicity of the molecules. An abnormally high relativistic correction was observed for phosphorus in PN, reaching ~20% of the CCSD(T)/CBS value, while the correction was less than 7% for other tested molecules.

## 1. Introduction

Computed NMR parameters are often used to support experimental observations or to predict properties of new compounds. Thus, accurate theoretical predictions of components of nuclear magnetic shieldings, isotropic shielding constants (and chemical shifts, respectively) and shielding anisotropies have always been in great demand [1,2,3,4]. The experimental nuclear shielding tensor characterizes the response of a local nuclei to an external magnetic field. Nowadays, theoretical methods allow the prediction of this absolute parameter. In contrast, the experimental observable, chemical shift is a relative parameter, which requires a reference signal. The quality of predicted NMR shieldings depends on various theoretical aspects [1,3,5]. The basis set type and size used in NMR shielding predictions are among the most important factors strongly affecting the quality of predicted values. In the case of a theoretical chemical shift, the results often benefit from accidental error cancellation [1,6,7,8]. In general, the calculated NMR shieldings are sensitive to a proper description of electrons and they improve with the completeness and the flexibility of the basis sets. The atomic nuclei are shielded by both valence and core electrons, which interact with the external magnetic field, inducing a magnetic field in the opposite direction. Thus, any reliable GIAO NMR calculation [9,10] should properly describe both types of electrons. The typical approach in the calculations of shieldings is that a series of basis sets with well-defined quality levels is employed for calculations that, in favorable cases, allow the extrapolation of results to the CBS limit [11,12]. However, the CBS limit of the ^1^H, ^13^C, ^15^N and ^17^O NMR shieldings is achievable only for small- or medium-sized isolated molecules [13,14,15], but yet it is out of reach for larger molecules. Note that generally good results are obtained with dedicated basis sets, optimized for specific methods and properties [16,17,18].

To aid thermochemical calculations of energy and energy-related parameters performed with relevant basis sets, various basis set families have specifically been designed for accurate predictions of GIAO NMR parameters. For example, the Dunning correlation-consistent (aug)-cc-pV*X*Z basis set [19,20,21,22,23], where *X* = D, T, Q, 5, and 6, was designed to treat reliably and efficiently electron correlation between the valence electrons. The further extension (augmentation) was then used to specifically treat the polarization due to an external electric field. Properties of correlation-consistent basis sets are also reported in detail [19,20,21,22,23]. Later, they were modified to also include core-valence electrons, giving rise to the (aug)-cc-pCV*X*Z, and aug-cc-pwCV*X*Z basis sets [21,24,25]. Similarly, Jensen polarization-consistent basis set families (aug)-pc-*n* [26,27,28,29], where *n* = 0, 1, 2, 3, and 4, were designed and used for accurate calculations of energy and energy related properties (originally for SCF-HF and DFT). The characteristic feature of these basis set families is an exponential-like decrease in energy of an atom or molecule as a result of calculation according to increased cardinal number *X* (or *n*). Later, Jensen designed the aug-pcS-*n*, aug-pcSseg-*n* and aug-pcJ-*n* [17,30] basis set families for efficient predictions of nuclear shieldings and indirect spin–spin coupling constants. It is generally accepted that GIAO NMR parameters calculated with the smaller Dunning basis set (significantly truncated for lower *X*) are inferior to those obtained with the larger basis set [4,31,32]. Thus, it is expected that the NMR parameters improve significantly from *X* to *X* + 1 in a regular way as was documented in [4,31,32,33]. In a seminal review on the calculation of nuclear shieldings and coupling constants, Helgaker, Jaszunski, and Ruud [1] noticed some deficiencies of the cc-pV*X*Z basis set series and proposed their improved version, with the inclusion of core-valence treatment (cc-pCV*X*Z) in future studies.

Another hierarchy of basis sets, primarily developed by Ahlrichs and coworkers [34], are the so-called Karlsruhe x2c-Def2 basis sets. Despite their compact size, these basis sets have recently been recommended for accurate calculations of nuclear shieldings [35,36,37]. The Karlsruhe x2c-Def2 basis sets are also suitable for the treatment of scalar relativistic effects but are smaller than Douglas–Kroll modifications of the Dunning type [38].

Most correlated calculations of NMR shieldings are performed with a focus only on valence electrons but core electrons become important even for moderately heavier NMR-active nuclei, such as ^27^Al, ^31^P, and ^33^S. Indeed, neglecting core electrons could perturb a regular convergence of NMR parameters toward the complete basis set limit, which is observed for ^1^H, ^13^C and ^15^N. A completely different picture was observed recently for the ^31^P shielding constants in the phosphorus mononitride (PN) molecule [15]. In this case, the phosphorous isotropic shielding σiso (and similarly shielding anisotropy, Δσ) calculated with the SCF-HF, DFT-KT3, MP2, CCSD and CCSD(T) methods, and combined with the (aug)-cc-pV*X*Z basis sets, were scattered, evincing nonstandard convergence with increasing basis set size. In addition, the scatter patterns were very similar for all the studied methods. Going from double- to triple-ζ, the ^31^P isotropic shielding in PN calculated with the CCSD(T) method dropped by approximately 190 ppm and then went back up by 20 ppm for the quadruple-ζ basis set and again decreased by 70 ppm with the quintuple-ζ basis set. Finally, a saturation of phosphorous isotropic shielding was observed for *X* = 5 and 6. At the same time, regular exponential decreases in total energy as well as the ^15^N isotropic shielding were observed.

The importance of including the core-valence basis sets in accurate calculation of nuclear shieldings has been demonstrated in several wave-function and density-functional studies [39,40,41]. For example, Field-Theodore et al. [40] studied nuclear shieldings of NF_3_, PF_3_, and AsF_3_ using all-electron CCSD(T) calculations with the valence and core-valence basis sets. However, there is no clear general picture of regular nuclear shielding convergence for the third-row nuclei upon improving the cc-pV*X*Z or cc-pCV*X*Z basis set series in the literature. We can find some studies on selected third-row nuclei, though. Recently, a HF/aug-cc-pV*X*Z study on ^27^Al NMR chemical shift of Al(OH)_4_^−^ appeared [42], showing a similar scatter of ^27^Al nuclear isotropic shielding upon increasing the cardinal number of Dunning basis set, as was observed for PN [15]. The calculated values in the series differed by −48 ppm when changing *X* from D to T, by +20 ppm when going from T to Q, and finally by −20 ppm for the changes from Q to 5. The authors modified the standard aug-cc-pVQZ Dunning basis set by the addition of a tight p-function and the scatter of the calculated nuclear shieldings disappeared. Interestingly, the use of the core-valence aug-cc-pCV*X*Z basis set family produced regularly converging ^31^P NMR parameters in PN [15]. Unfortunately, no analysis of ^31^P shielding components in PN was performed in the work [15]. These parameters should be more sensitive to the basis set quality than the total isotropic value which is calculated as one-third of the trace average of the nuclear magnetic shielding tensor. Further, no direct comparison of the convergence patterns of the ^31^P NMR shieldings in small molecules has been published so far. From the aforementioned literature compilation, it is apparent that a systematic test of nuclear shieldings convergence for third-row elements using the SCF-HF, B3LYP, and CCSD(T) methods combined with the aug-cc-pV*X*Z, aug-cc-pCV*X*Z, aug-pcSseg-*n*, and Karlsruhe-type basis sets has not been reported.

Given all the above, there is still an open question that is the behavior of calculated NMR shieldings obtained with the aug-cc-pV*X*Z basis set for all chemical elements in the third row of periodic table, namely Na, Mg, Al, Si, P, S, Cl and Ar. NMR spectroscopy for ^25^Mg, ^35^Cl and ^39^Ar (a radioactive isotope) is not common. Most of the NMR-active nuclei from the third row are quadrupolar, with a spin of 3/2 (^23^Na, ^33^S, ^35^Cl, or ^37^Cl) or 5/2 (^25^Mg, ^27^Al), and therefore their signal broadens with asymmetry of the environment. Only ^29^Si and ^31^P have ½ spin. The sensitivity of the aforementioned nuclei is from low (^25^Mg and ^33^S) to medium (^23^Na, ^29^Si and ^31^P) or even high (^27^Al). ^35^Cl is more sensitive than ^37^Cl; on the contrary, ^37^Cl provides a slightly higher resolution than ^35^Cl. Therefore, ^35^Cl is usually preferred over ^37^Cl. In general, quadrupolar nuclei have broader signals than silicon and phosphorus (1/2 spin) that yield sharp lines. The main use of sodium, magnesium or aluminum NMR is to determine their presence, or to monitor their binding, e.g., to biomolecules (Mg). Silicon NMR is mainly applied in material science, battery materials, civil engineering, or geology as the solid-state ^29^Si NMR [43]. Since ^31^P is a naturally abundant active nucleus that is more sensitive than ^13^C or ^15^N, it was utilized in a wide range of fields, such as cellular biochemistry, metabolomics, medicine, or synthetic chemistry [44]. Ultimately, accurate theoretical calculations for these isotopes could complete the picture about the sensitivity of predicted NMR features (e.g., shieldings) to individual computational approaches (e.g., to increased basis set cardinal number *X*). As result, this knowledge could help to design reliable, accurate, but simplified tools for simulation of a nuclear shielding tensor for these elements. 

We need to mention that the quality of gauge-including atomic orbital (GIAO) NMR calculations of shieldings is generally also sensitive to the description of the electron correlation. Thus, the precision of methods commonly used for shielding predictions decreases in the following order: CCSD(T)>MP2≈DFT>SCF−HF. Since the DFT approximation may often lead to a comparable quality of results as computationally more demanding MP2, the choice of a particular density functional from their great variety is also of prime importance [1,4]. Additionally, predicted NMR shieldings are often improved by inclusion of the zero-point vibrational correction (ZPVC) [45,46], the temperature correction (TC) [32,45,46], and also relativistic corrections (RCs) when the system contains heavy atoms [47,48,49,50]. For practical reasons, the solvent effect needs to be considered often to allow a direct comparison of calculated NMR parameters with experiment, mainly conducted in solution. 

Finally, we want to clarify some issues related to the use of estimated theoretical nuclear shieldings at the CBS limit and experimentally determined gas-phase chemical shifts (and nuclear shieldings). Obviously, the CBS shielding values are only values obtained by simply fitting the data obtained for a set of consecutive incomplete basis sets. Thus, the quality of any computational method, for example HF, DFT or CCSD(T), in predicting gas-phase or solution-phase NMR properties could be assessed from an error of the CBS value from experiment, when all aforementioned effects are considered (the ZPVC, the TC, RCs, and the solvent effect). According to earlier studies, the quality of theoretical predictions of experimental chemical shifts also depends on the following factors: (1) selection of a proper (similar) reference compound [6,51,52]; (2) statistical treatment of possible conformers, especially those stabilized by intramolecular H bonds [7,8]; and (3) proper inclusion of an explicit solvent effect, especially for polar protic solvents [51,52]. In the latter case, the compromise of including only the first solvation/hydration sphere is useful. This can significantly change the order of various signals in the theoretically predicted NMR spectrum and accurately reproduce the experimental image [53].

The aim of this study is to find a simple remedy to improve the irregular convergence patterns towards CBS of nuclear shielding tensors of simple molecules (or atoms) preferentially containing the third-row elements, calculated with the aug-cc-pV*X*Z basis set families, leading to the exponential-like behavior of the calculated NMR parameters. All benchmark NMR calculations were performed for free molecules in the gas phase and the results were compared with available experimental data (the solvent effect was not considered). However, ZPV, TC and RC corrections were included for direct comparison of theoretical results with experiment.

## 2. Results

For the convenience of the reader, we will use several abbreviations, including basis sets, instead of their full names in the following sections. All abbreviations are summarized in Table 1.

### 2.1. Sensitivity of Total Shieldings of the Third-Row Nuclei to the Basis Set Quality

We have recently reported the irregular basis set convergence of theoretical ^31^P isotropic shieldings of PN, calculated using the Hartree–Fock, but also DFT and coupled-cluster methods [15]. We obtained such irregularity for Dunning aug-cc-pV*X*Z or aug-cc-pV(*X*+d)Z basis sets, while a smooth convergence was observed for the core-valence aug-cc-pCV*X*Z basis set. On the other hand, we achieve a smooth basis set convergence of ^15^N NMR shieldings in PN with all the basis set families [15]. We decided to inspect whether the irregularity observed for ^31^P in PN is a general behavior of all elements of the third row. Therefore, we extended our test set by small systems (mainly hydrides) containing magnetic active nuclei of the third row, namely ^23^Na, ^25^Mg, ^27^Al, ^29^Si, ^31^P, ^33^S, ^35^Cl, and ^39^Ar as a free atom, and calculated their isotropic shieldings using various methods and basis set families. We will discuss most of the findings of an important NMR nucleus, ^31^P, as an example, using the three model molecules. The results for other third-row nuclei will be briefly summarized afterwards.

#### 2.1.1. Sensitivity of ^31^P NMR Parameters to the Basis Set Quality

As model compounds for a thorough analysis of ^31^P nuclear shieldings, we have selected systems where phosphorus is joined to other atoms by a single or multiple bond. A popular hydride, PH_3_ contains only three single bonds. As a system with a double bond, we picked up phosphine oxide, H_3_PO. We also extended our recent study on another molecule, PN, present in the interstellar space [15]. Note that the bond in PN, a molecule also briefly discussed here, is not strictly speaking a triple bond, but rather something between a double and a triple bond [15].

For brevity, most individual data discussed in this section are gathered in tables and figures in the Supplementary Material, as indicated in the text.

**Figure 1:**
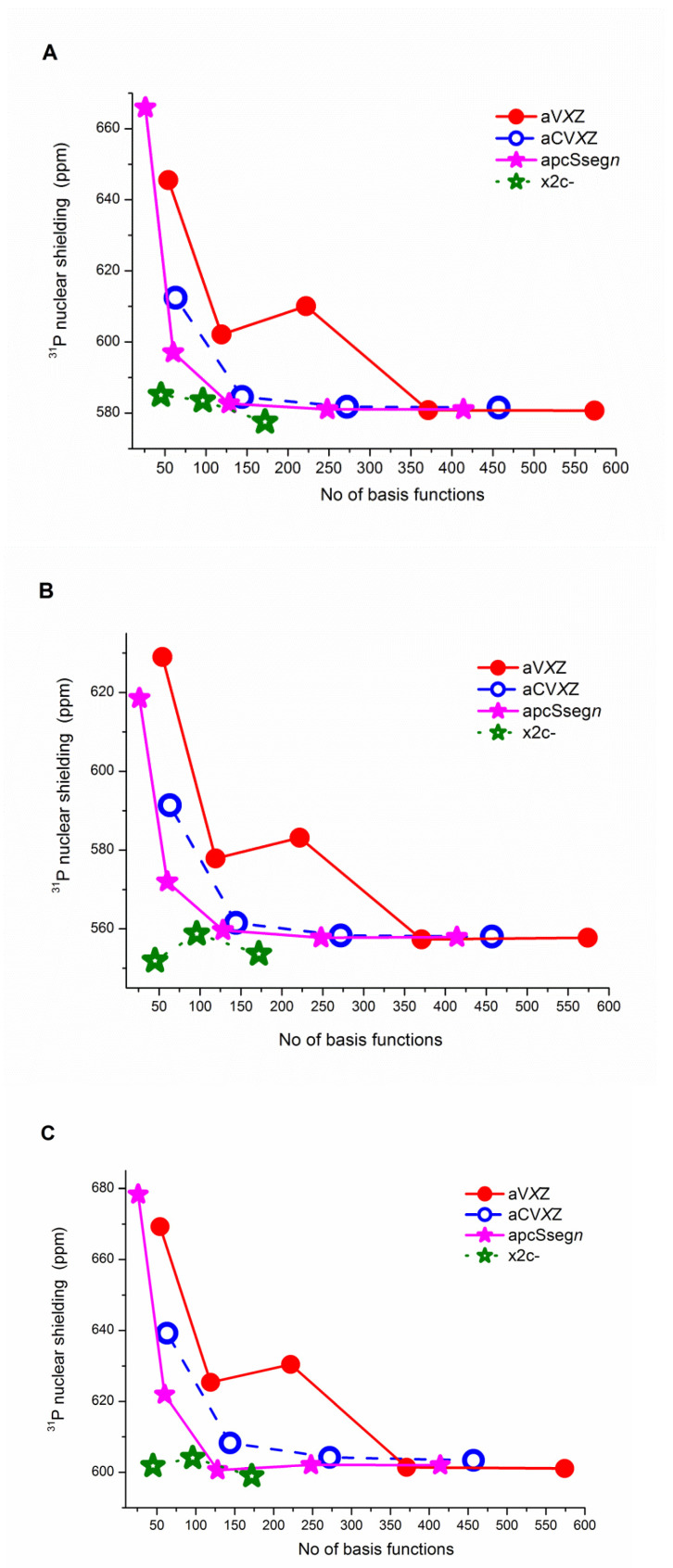
Convergence of ^31^P isotropic shielding constants for PH_3_ vs. the number of basis functions, calculated with the (**A**) HF-SCF, (**B**) B3LYP and (**C**) CCSD(T) methods combined with the aug-cc-pV*X*Z, aug-cc-pCV*X*Z, aug-pcSseg-*n* and x2c-*X*ZVPall-s basis set families.

First, individual ^31^P nuclear shielding values for PH_3_, calculated with the SCF-HF, B3LYP and CCSD(T) methods and with four selected basis set series (the aug-cc-pV*X*Z, aug-cc-pCV*X*Z, aug-pcSseg-*n* and x2c-*X*ZVPall-s basis set families) are gathered in Table S1A. Total energies of phosphine calculated at the B3LYP and CCSD(T) levels with two selected correlation-consistent basis sets are included in Table S1B. In Figure S1, we can see a regular convergence of the B3LYP/aug-cc-pV*X*Z and B3LYP/aug-cc-pCV*X*Z energies for PH_3_ towards the complete basis set limit. On the contrary, Figure 1 displays quite irregular convergence patterns of NMR shielding constants for ^31^P in PH_3_, calculated with the HF-SCF, B3LYP, and CCSD(T) methods and the aug-cc-pV*X*Z basis set, unsuitable for any extrapolation with more than than two-points to the CBS limit. Calculated isotropic shieldings (obtained, e.g., at CCSD(T)/aug-cc-pV*X*Z; cf. Table S1A) change upon increasing the basis set size by: Δ = −44, 5, −29, and 0.3 ppm, respectively, where Δ is calculated for T →D, Q→T, 5→Q, and 6→5. On the other hand, shieldings obtained with the Dunning core-valence basis set family smoothly converge towards the CBS limit, following the exponential decay curve (e.g., see converging HF-SCF data for X = T, Q and 5 in Figure 1A). The estimated CBS ^31^P isotropic nuclear shielding, calculated at the CCSD(T)/aug-cc-pCV*X*Z level of theory in PH_3_ is 603.326 ppm (see Table S1A). When vibrational, thermal, and relativistic corrections were included (see Table 4), we obtained the final CCSD(T) value of 611.38 ppm. Apart from aug-cc-pCV*X*Z, the Jensen aug-pcSseg-*n* basis set hierarchy also yields a regular and smooth convergence (see Figure 1) of phosphorus shielding towards the CBS limit (580.892 ppm for HF-SCF, 557.661 ppm for B3LYP, and 588.578 ppm for CCSD(T)). It is important that these results are nearly converged already for aug-pcSseg-2, with only 128 basis functions. The last tested basis sets are from the Karlsruhe family. These basis sets are relatively small (45, 96 and 172 basis functions for x2c-SVPall-s, x2c-TZVPPall-s, and x2c-QZVPPall-s, resp.). All of these basis sets provide shieldings that are fairly close to the CBS limit estimated with the aug-cc-pCV*X*Z and aug-pcSseg-*n* series (see Figure 1 and Table S1A). The approximate CBS limit, estimated using the Karlsruhe basis sets, is 601.348 ppm (the CCSD(T) level). Nevertheless, we cannot call the convergence smooth in this series due to the small number of values.

To extend our recent study on ^31^P NMR shieldings of PN [15], we performed here additional DFT-B3LYP calculations to also determine the sensitivity of individual shielding components, isotropic and anisotropic shieldings of P and N nuclei to the selected basis sets and their size (Tables S2–S4) if calculated at the DFT level. In Figures S2 and S3, the B3LYP/aug-cc-pV*X*Z-calculated ^31^P shielding constants and their components are shown according to the cardinal number *X* (Figure S3A) and the number of basis functions (Figure S3B). Note that the shielding convergence patterns plotted against *X* or the number of basis sets are essentially the same. We observed significantly scattered results obtained with aug-cc-pV*X*Z basis for *X* = D, T and Q, and only the results for *X* = 5 and 6 seem to converge to the complete basis set limit.

The analysis of components suggests that the main source of the irregularity originates in the paramagnetic contribution of the nuclear shielding. We inspected the diamagnetic (DSO) and paramagnetic (PSO) contributions to total phosphorus shielding in PN calculated at the B3LYP level with the aug-cc-pV*X*Z, aug-cc-pCV*X*Z, aug-pcSseg-*n*, aug-pc-*n*, and aug-pcJ-*n* basis sets (Table S2B). We can see that while DSO calculated with the aug-cc-pV*X*Z basis set converges relatively smoothly, PSO exhibits the scattering of data. As expected, the results obtained with the aug-cc-pwCV*X*Z basis set family, better describing the core-valence electrons, provide less scattered shielding components upon increasing the basis set size. The shielding components calculated with the latter basis set family are also more regularly converging toward the CBS limit. For brevity, similar correlation patterns of ^31^P nuclear shielding components in PN, obtained with aug-cc-pCV*X*Z, aug-cc-pwCV*X*Z, aug-pc-*n*, aug-pcJ-*n* and aug-Sseg-*n* basis sets vs. *X* are shown in Figure S3A in the Supplementary Material (see also Table S2A). No matter whether we plot the shielding components against *X* or the number of basis functions, the same convergence pattern was observed (Figures S2 and S3), only for the latter case, the size of individual basis sets is more imaginable. Note the break on the *y*-axis and different scaling for individual components in Figures S2 and S3; thus, the σ_xx_ component varies by approximately 160 ppm, while σ_zz_ changes only by approximately 1 ppm.

It is also apparent from Figures S2 and S3 that the NMR results obtained with the aug-cc-pV*X*Z basis sets are highly scattered and unreliable for *X* = D, T and Q and evince saturation for *X* = 5 and 6. On the other hand, the results obtained with the core-valence basis sets (aug-cc-wCV*X*Z) regularly converge within the whole set, reaching values close to CBS already for aug-cc-wCVTZ. Thus, highly reliable ^31^P shielding components for larger system can be achieved with properly chosen core-valence triple-ζ basis set. Considering the performance of Jensen basis sets, all available series converge exponentially, perform fairly well and the CBS values are within ±5 ppm from each other (see Table 2 and Figure S4). Obviously, results obtained with Jensen basis sets using too small values of n (0 and 1) are unreliable. Finally, one can observe that the Douglas–Kroll modification of polarized-valence Dunning-type basis sets also produces scattered ^31^P shieldings for PN (Figure S5).

Detailed comparison of the B3LYP/CBS values of ^31^P NMR parameters obtained for PN with selected basis set families are in Table 2. In general, the CBS values, estimated according to *X* (see Table S4) or the numbers of basis functions (Table 3), are very similar and differ by less than 1%. Only in the case of isotropic shielding calculated with the aug-cc-pV*X*Z and aug-pcJ-*n* basis set families are the differences slightly larger (4.4 and 7.0%). Corresponding ^15^N CBS values of PN are significantly closer to each other and differ by less than 0.5%. Gathered NMR parameters are also compared with earlier reported values. As expected, the ^31^P nuclear isotropic shielding calculated at the B3LYP level is nearly 120 ppm smaller than the CCSD(T) results, but the shielding anisotropy is approximately 180 ppm larger. Obviously, the DFT methods usually do not provide reliable predictions of ^31^P NMR parameters [4]. However, in the current study, we aimed at converged results close to the CBS limit for a selected method and at the behavior of calculated values with increasing basis set, rather than at precise predictions of a particular NMR property.

Similar to PN, we wanted to inspect whether an analogously irregular convergence pattern of ^31^P shielding is also obtained for other P-containing molecules with multiple bonds when the aug-cc-pV*X*Z family is used. Phosphine oxide includes a double P=O bond and their shielding differs markedly from PH_3_. For brevity, the individual ^31^P nuclear shieldings for H_3_PO calculated at the B3LYP, HF, and partially also at the CCSD(T) level with the aug-cc-pV*X*Z, aug-cc-pCV*X*Z, and aug-pc-Sseg-*n* basis sets are gathered in Table S5A and shown in Figure S6. Once again, the nuclear shieldings obtained using Dunning valence basis sets are scattered and do not follow a smooth convergence pattern. On the contrary, NMR parameters predicted with polarized-consistent basis sets show regular convergence. Due to convergence problems, we did not obtain the full series of the CCSD(T) values for all basis sets (see Table S5A). However, it is already evident that ^31^P shieldings at HF match the CCSD(T) values.

#### 2.1.2. Other Third-Row Elements

On concluding the analysis of ^31^P nuclear shieldings convergence in selected systems, we will perform a brief analysis of nuclear shieldings for other third-row elements. We start with the ^23^Na NMR parameters of NaH calculated using the Hartree–Fock, B3LYP, and CCSD(T) methods and various basis sets. In addition, we calculated ^23^Na nuclear shieldings for NaF using the B3LYP hybrid function combined with the aug-cc-pVXZ, aug-cc-pCVXZ, and aug-pcsSeg-*n* basis set families. Then, we will move to hydrides of the remaining elements calculated at various levels of theory as indicated in the text. The tests will end with hypothetical NMR parameters predicted for an isolated argon atom. Indeed, the NMR-active ^39^Ar isotope does not exist in nature but analysis of its hypothetical NMR parameters will complete the GIAO NMR studies of the third-row elements. Obtained results will be commented only briefly, but the fully detailed text is in Supplementary Materials (Sections S1.1–S1.7).

Before we analyze all calculated shieldings, we tested the eligibility of the B3LYP geometries in further calculations of NMR shieldings of the third-row elements. NaH served us as a model system. First, we calculated interatomic distances of NaH at the B3LYP level with the cc-pVTZ and cc-pCVTZ basis sets and compared them with the CCSD(T) geometries obtained with the same basis sets. Reported [56] distances for the CCSD(T)/cc-pVTZ and CCSD(T)/cc-pCVTZ levels were 1.916 A° and 1.893 A°, respectively. We observed slightly shorter distances for the B3LYP but also for the CCSD(T)—1.8832 A° (B3LYP/aug-cc-pVTZ), 1.8821 A° (B3LYP/aug-cc-pCVTZ), 1.8801 A° (CCSD(T)/aug-cc-pVTZ), and 1.8947 A° (CCSD(T)/aug-cc-pCVTZ). Later, we obtained Na–H distances of 1.8808 A° and 1.8811 A° at the B3LYP/aug-cc-pV5Z and B3LYP/aug-cc-pCV5Z levels that are very close to the CCSD(T) values. These values are also close to the experimental distance of 1.8874 A° [56]. Subsequent estimations of ^23^Na nuclear shieldings at the B3LYP/aug-cc-pV5Z level of theory for the B3LYP/aug-cc-pV5Z and B3LYP/aug-cc-pCV5Z geometries provide similar values of 559.698 and 559.729 ppm. Note also that the calculated CCSD(T) nuclear shieldings achieved for the DFT and CCSD(T) geometry (quintuple-ζ basis set) were very close (541.910 and 540.964 ppm, respectively). Thus, the B3LYP/aug-cc-pV5Z geometries appear to be good estimates achievable relatively easily and will be used to gain 3D structures of several other model compounds (NaH, NaF, MgH_2_, AlH_3_, and HCl) in this study. However, for the sake of comparison with earlier studies, the PN, PH_3_, SiH_4_ and H_2_S geometries from recent reports [15,56,57] were used.

The ^23^Na nuclear shielding values calculated at the B3LYP/aug-cc-pV*X*Z and B3LYP/aug-cc-pCV*X*Z levels for NaH are gathered in Table S6A. For a better perspective, the convergence patterns of ^23^Na isotropic shieldings of NaH calculated at the HF-SCF, B3LYP-DFT and CCSD(T) levels of theory and with the aug-cc-pV*X*Z, aug-cc-pCV*X*Z, aug-pcSseg-*n* and x2c-Def2 basis set families are shown in Figure S7. Similarly to ^31^P, the aug-cc-pV*X*Z series produces scattered ^23^Na isotropic shieldings, while a smooth convergence is seen for the aug-pcSseg-*n* family. Interestingly, despite their small size, the x2c-Def2 basis family performs fairly accurate in comparison to Jensen or core-valence basis. We observed the analogous behavior of ^23^Na shieldings for another sodium-containing molecule, NaF (Table S6A, Figure S8). Note that energies for all calculated sodium models exhibit presumed exponential patterns (see Table S6B, Figure S9).

We observed analogous basis set convergences of isotropic shieldings also for ^25^Mg (MgH_2_), ^27^Al (AlH_3_), ^29^Si (SiH_4_), ^33^S (H_2_S), and ^35^Cl (HCl). In all cases, the Dunning basis set family provided irregular convergence patterns, whereas other tested basis sets behave as expected, giving smooth (exponential-like) patterns. All isotropic shieldings calculated at various levels are summarized in Table S7A (MgH_2_), Table S8A (AlH_3_), Table S9A (SiH_4_), HSi≡CH [58] (Table S9C), Table S10A (H_2_S), and Table S11A (HCl). For a better idea of different convergences, we plotted the CCSD(T) shieldings obtained with different basis sets in Figure S10A (MgH_2_), Figure S11A (AlH_3_), Figure S12A (HF-SCF results for SiH_4_), Figure S13A (H_2_S) and Figure S14A (HCl). In Figure S12C, the results of B3LYP calculations with aug-cc-pV*X*Z and aug-pcSseg-*n* basis sets for ^29^Si nuclear shieldings in the Hsi≡CH molecule are graphically presented. Corresponding energy estimates of all systems are gathered in Tables S7B, S8B, S9B, S10B, and S11B or Figures S10B, S11B, S12B, S13B and S14B. Similarly to phosphorus, all tested basis sets provided smooth convergence patterns of estimated energies (all systems) with increasing basis set size. Interestingly, the difference between shieldings calculated using the highest Dunning basis set (aug-cc-pV5Z) and the lowest basis set (aug-cc-pVDZ) usually depends on the level of theory. For example, we observe for ^25^Mg (MgH_2_) the difference of −35, −98 and −73 ppm for HF-SCF, B3LYP, CCSD(T), respectively. Since the ^25^Mg CBS value is approximately 400 ppm (see Table 3), this means that these differences account for 8 to 25% of the CBS value. As indicated above, the differences between values calculated with consecutive Dunning basis sets change unpredictably producing a scattered convergence pattern. For example, ^27^Al isotropic shieldings in AlH_3_ calculated at the CCSD(T)/aug-cc-pV*X*Z level vary by −74, −4, −15 ppm for *X* = T→D, Q→T and 5→Q, respectively. The absolute changes (but also relative to the CBS value) according to increasing basis set size depend on the individual methods (see Tables S7A, S8A, S9A, S10A and S11A, and Sections S1.1–S1.7 for more comments on individual data).

Table S12A analogously summarizes NMR shieldings for isolated Ar, as calculated using different methods and basis sets. Figure S15A then depicts the shielding convergence patterns corresponding to the different basis sets. Contrary to the aforementioned hydrides, we can see a fairly regular convergence pattern of ^39^Ar shielding even for the aug-cc-pV*X*Z family. Changes in the shielding with increasing basis set size are rather cosmetic (less than 0.1% of the CBS value). On the other hand, smaller Karlsruhe basis sets provided ^39^Ar shieldings more distinct from the CBS value resulting thus in an unusually scattered convergence pattern. Nevertheless, even the highest change between consecutive shieldings does not exceed 1% of the CBS value. Estimated energies for Ar are gathered in Table S12B and Figure S15B revealing their standard convergence for all methods and basis sets.

#### 2.1.3. Estimated CBS Nuclear Shielding Values of the Studied Systems

As documented in most figures (see, e.g., Figure 1, Figures S5, S6 or Figure S7), theoretical isotropic shielding constants for individual third-row nuclei estimated with double-ζ quality basis sets are problematic. The aug-cc-pVDZ values are far from the fitted CBS values and also from the convergence trendline estimated using the triple-ζ, quandruple-ζ, and quintuple-ζ basis set. Although the aug-cc-pCVDZ results more or less follow trends estimated using larger basis sets, they are still far from the convergence. Therefore, any reasonable CBS isotropic shielding of the third-row elements should be estimated excluding the double-ζ data. The CBS nuclear shieldings estimated using the 2-parameter fit of values obtained with the aug-cc-pV*X*Z and aug-cc-pCV*X*Z basis sets at the HF-SCF, B3LYP, and CCSD(T) levels are summarized in Table 3. The table also reveals the CBS values estimated using the Jensen basis sets. Corresponding CBS shieldings for the Karlsruhe x2c-*X*ZVPPall-s basis sets can be found in Tables S5A–S12A. 

We can observe a difference of nuclear shieldings calculated with the core-valence and valence basis sets. Nevertheless, the difference does not exceed for any molecule and any method 7% of the CBS(aug-cc-pCVXZ) value. This difference is due to the overestimation of shieldings obtained with smaller aug-cc-pV*X*Z (*X* = D, T, and even Q) basis sets that as a result deteriorates the finals CBS(aug-cc-pV*X*Z) value, as documented in Tables S5A–S12A. Therefore, we can consider the results obtained with the aug-cc-pV*X*Z basis set family less reliable. On the other hand, both basis set families produced very similar results for isolated argon atom. 

The impact of the electron correlation on calculated nuclear shieldings of the third-row elements can be clearly demonstrated by comparing the HF-SCF and CCSD(T) values of the molecules studied (see the Δ values in Table 3). The most significant discrepancy between the HF and coupled cluster results is observed for PN (~250%) and Hsi≡CH (>40%). In both compounds, the element of interest is bonded by a triple bond (the PN bond is, strictly speaking, something between a double and triple bond). This Δ is comparable for all tested basis set families. A significant deviation is also observed for AlH_3_ (approximately ~13%). We can assume that the importance of electron correlation for accurate prediction of nuclear shieldings of the third-row elements increases, especially when these atoms are bonded to other atoms by a multiple bond. On the other hand, we were not able to achieve the full set of the CCSD(T) shieldings for H_3_PO (due to convergence problems) that would confirm this assumption. However, partial data show that HF provides shieldings very close to CCSD(T), in contrast to B3LYP with an average error of ~10%. As observed before, the differences of the CBS values for the Hartree–Fock, B3LYP, and coupled cluster methods are negligible for argon [32]. Interestingly, the Δ compared for HF-SCF and B3LYP indicates the slightly better performance of the Hartree–Fock method than B3LYP for saturated molecules. This could be due to overestimation of paramagnetic term of shielding by B3LYP (see [4,59,60]).

### 2.2. ^33^S shielding Components and Total Shielding of 2-Thiouracil (2-TU) 

So far, we have mostly discussed systems where the heavy element was bonded with hydrogen (with the exception of HSiCH, PN, and H_3_PO). In this section, we will deal with a more realistic molecule containing a third-row element and other heavy atoms. Uracil is an important component related to information transfer and replication in living systems. Its 5-halogen modifications are used in anticancer and antifungal treatment [59,61,62,63]. Modifications of uracil, including replacing the oxygen atom with sulfur [64], also changes its biological activity. The presence of a sulfur atom can be exploited to easily identify the molecule by ^33^S NMR. Therefore, accurate theoretical predictions of sulfur shielding tensor in 2-thiouracil (2-TU) are valuable to help clarify the structure-spectrum relationship. The size of this molecule makes NMR calculations at the CCSD(T) level unavailable; therefore, all calculations had to be performed at the DFT (B3LYP) level as a reasonable compromise. The previously reported use of a relatively small basis set [65] (6-31G* containing 132 basis functions) for all atoms allowed fast but inaccurate calculations of nuclear shieldings of 2-thiouracil. To improve calculation reliability, we will show the effect of the locally dense basis set (LDBS) approach [66,67,68,69], where only the atom of interest is described with a higher basis set, while the rest of the molecule is described with some low-level basis set. Here, within the LDBS approach, all atoms were calculated with the 6-31G* basis set, while the aug-cc-phisV*X*Z basis set families are employed for sulfur (see Table S10C in Supplementary Materials). Note that the complete description of 2-thiouracil with, e.g., a very reliable aug-cc-pCV5Z basis set containing 1804 basis functions, lies beyond any practical use. On the other hand, the proposed LDBS approach with, e.g., aug-cc-pCV5Z/6-31G* basis sets, requires only 323 basis functions. 

**Figure 2:**
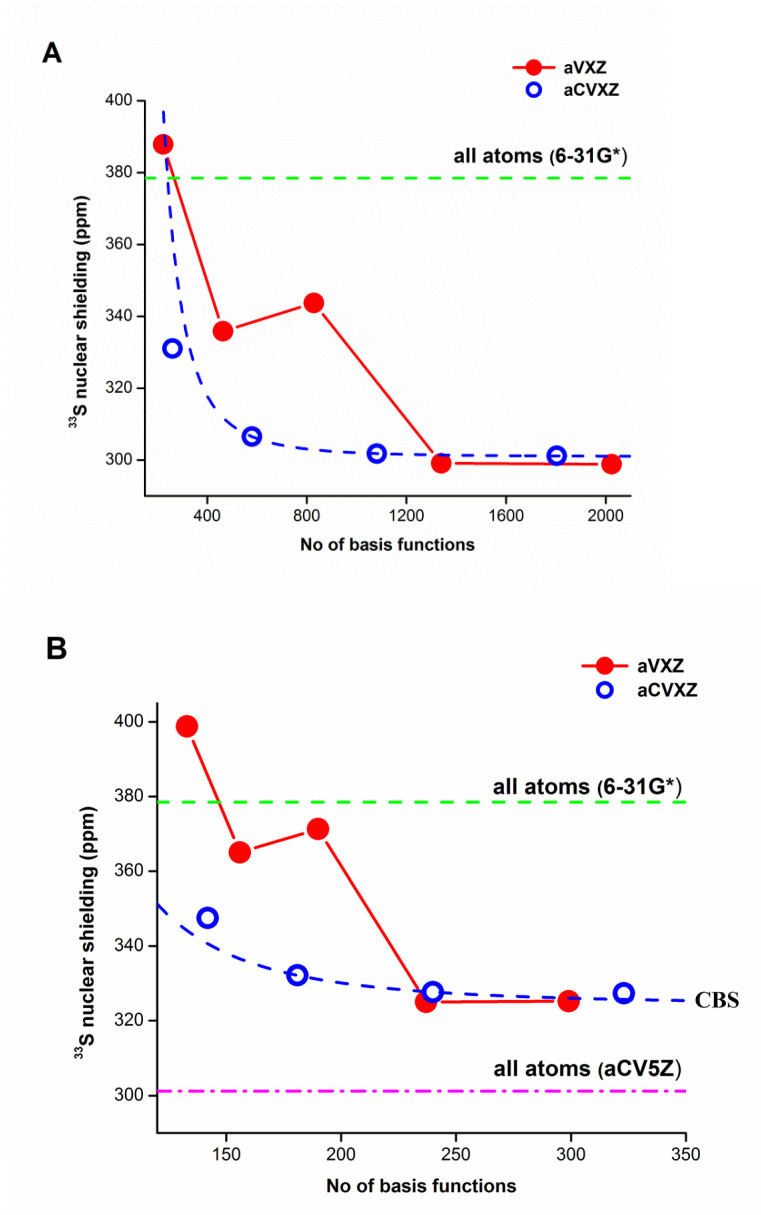
^33^S nuclear isotropic shielding constants of 2-thiouracil calculated at the (**A**) B3LYP/aug-cc-pV*X*Z and B3LYP/aug-cc-pCV*X*Z levels of theory, and (**B**) using the LDBS approach, where only the sulfur atom was described using either the aug-cc-pV*X*Z or aug-cc-pCV*X*Z and 6-31G* basis set on H, C, N and O atoms. The green dashed line indicates the value obtained at the B3LYP/6-31G* level. The pink dash-and-dot line on the right shows the value obtained at the B3LYP/aug-cc-pCV5Z level (all atoms).

The corresponding ^33^S isotropic shielding estimated by the B3LYP/6-31G* calculation was 335 ppm compared to 258 ppm obtained at the B3LYP/aug-cc-pCV5Z level. The total CPU time for the former level was 3.5 min and was almost 20 days for the latter. Using the LDBS approach (aug-cc-pCV5Z/6-31G*), we obtained the ^33^S isotropic shielding of approximately 287 ppm (see Figure 2), which is much closer to the full aug-cc-pCV5Z value of 258 ppm. At the same time, a very impressive reduction in CPU time was observed, which dropped to 27.5 min. Figure 2 also compares convergence patterns of the ^33^S isotropic shieldings for 2-TU calculated at the B3LYP level with the aug-cc-pV*X*Z and aug-cc-pCV*X*Z basis sets. For the first family of basis sets, we observe a scatter of calculated values comparable to H_2_S, while a smooth convergence is evident for the latter. Same behavior can be noticed if we employ the LDBS approach (Figure 2B). The change in estimated CBS values due to the LDBS approach is ~10% of the CBS value when all atoms were described using aug-cc-pV*X*Z or aug-cc-pCV*X*Z.

### 2.3. Corrections to Isotropic Nuclear Magnetic Shieldings of Third Row Elements 

We compared our best isotropic shieldings for all our molecules that were estimated using the CCSD(T)/aug-cc-pCV*X*Z data with available literature data in Table 4. It has been documented before that the equilibrium value (calculated for the optimized geometry) is often not precise enough, as vibrational effects, thermal effects, or relativistic corrections may become significant, especially for heavier elements [47,48,50,70,71].

Therefore, our CCSD(T)/CBS values were later corrected by the zero-point vibrational, thermal and relativistic corrections (the TC and the RC). Thus, the final values were obtained as: final value = equilibrium CCSD(T)/CBS σ + ZPVC + TC + RC. Our correction terms were also compared with available reported corrections (see Table 4). Note, there are no available equilibrium shieldings reported in the literature for NaH, MgH_2_, AlH_3_, H_3_PO, or HSi≡CH. Jaszunski et al. performed accurate coupled cluster estimates of nuclear shielding also considering the ZPVC and the TC for SiH_4_ [57], PH_3_ [55,57], H_2_S [57], and HCl [72]. The relativistic effects on the total shielding value were considered only for HCl [72]. Argon nuclear shieldings were studied by Hada [73]. Sauer et al. also estimated the relativistic contribution to the argon isotropic value [32].

We first discuss the TC and the ZPVC separately for hydrides, as the selected systems with multiple bonds (H_3_PO, HSi≡CH, and PN) appeared to be exceptional or even problematic. Note that the TC is negligible for all third-row element shieldings in the hydrides studied. The ZPVC estimated at the B3LYP/aug-cc-pVQZ level ranges from ~0.05% for the ^23^NaH to >4% for ^29^Si in SiH_4_. Other noticeable (>2%) ZPVCs were observed for MgH_2_, and H_2_S. All other ZPVCs were lower than 2% of the isotropic value. Interestingly, positive ZPVCs to isotropic shieldings were observed for NaH, MgH_2_ and SiH_4_, while negative values were obtained for AlH_3_, PH_3_, H_2_S, and HCl. Note that the ZPVC may depend on the level of theory used for calculating the anharmonic potential and shielding derivatives. Therefore, we alternatively calculated the anharmonic force fields at the CCSD(T)/aug-pc-2 level and combined them with shielding derivatives at the BHandHLYP/aug-pcSseg-4 level. A similar combination of different levels for the anharmonic potential and for the property derivatives has previously been shown as an economical approach for reliable estimations of ZPVCs of medium-sized molecules [32,41,74]. We noticed significant differences between the fully B3LYP and the mixed ZPVCs, especially for SiH_4_ (20.28 versus 1.85 ppm). Nevertheless, the higher B3LYP values still represent only ~2% of the total shielding value. In general, we consider the mixed approach closer to the fully coupled-cluster ZPVC value and thus likely more reliable for unsaturated molecules.

Predictions of the ZPVC for the selected molecules with multiple bonds, HSi≡CH, H_3_PO, and PN, appeared to be more interesting. Due to their nature, a standard perturbational approach used for calculating the ZPVC failed when some of the lowest vibrational modes were included in the PT2 formula. As a result, the unrealistic ZPVC or TC, as well as vibrational frequencies, were obtained. Therefore, we had to exclude the contribution of the two lowest vibrational modes for HSi≡CH, and even three lowest modes for H_3_PO. Even so, ZPVCs calculated using both methods (B3LYP and CCSD(T)/BHandHLYP) vary significantly. We obtained for example −0.69 ppm (B3LYP) and 30.77 ppm (CCSD(T)/BHandHLYP) for HSi≡CH. The ZPVC contribution thus makes −0.1% or 4.9% of the CCSD(T)/CBS equilibrium value. This discrepancy may be due to various reasons. For example, a different basis set convergence of the two methods, inadequate numerical steps in the ZPVC calculation, significant contribution of quartic constants, or more general failure of the methodology used. An even larger deviation is observed for H_3_PO, where we calculated the ZPVC of −4.72 ppm (B3LYP) and 69.39 ppm (CCSD(T)/BHandHLYP), which makes −1.4% and 20.0% of the equilibrium value. Note that we were not able to obtain the CCSD(T)/CBS equilibrium value, only the B3LYP/CBS, and therefore the final percentage contribution may change. On the other hand, ZPVCs for PN are comparable for both methods. We conclude that ZPVC calculations for compounds with double or triple bonds deserve further investigation and we can only speculate that the B3LYP values reported here represent more reliable estimates.

As expected, the absolute relativistic correction (see Table 4) increases with atomic mass ranging from ~8 ppm for Na to ~33 ppm for Cl and Ar. Nevertheless, the RC accounts for 1.4–6.5% of the CCSD(T)/CBS equilibrium shielding value for most systems. Two different levels of theory for the RC were tested, yet both DFT approaches provide in most cases comparable results (see Table 4). Interestingly, AlH_3_ and H_3_PO had a higher percentage relativistic contribution than H_2_S or HCl. The relativistic correction of PN was completely out of line with the others, accounting for 19% or 33% depending on the method. We estimated the basis set effect on the B3LYP RC for PN, where we obtained a larger contribution. The relativistic correction calculated at the B3LYP level and the aug-cc-pV*X*Z basis set, where *X* = D, T, and Q are summarized in Table S13. The double-ζ basis set provides obviously overestimated results, while the aug-cc-pVQZ value is close to the KT2/pcS-3 value. Nevertheless, the triple-ζ values may represent a good compromise between accuracy and price.

## 3. Discussion

Convergence patterns of the third-row elements nuclear shieldings were tested using the SCF-HF, DFT-B3LYP and CCSD(T) methods combined with the aug-cc-pV*X*Z, aug-cc-pCV*X*Z, and several polarization-consistent Jensen-type basis set series. The shieldings calculated with the aug-cc-pV*X*Z basis set family show an irregular convergence towards CBS. An erratic convergence of nuclear shieldings calculated with aug-cc-pV*X*Z (*X* = D-5 or 6) was observed for a test set of the simple molecules (NaH, MgH_2_, AlH_3_, SiH_4_, HSi≡CH, PH_3_, PN, H_3_PO, H_2_S, and HCl) and Ar atom studied. By improving the valence basis sets to core-valence, a regular (exponential) convergence of shieldings towards CBS could be observed. A similar improvement was also observed for shieldings calculated using the with Jensen-type basis set families. 

On the contrary, a relatively smooth convergence was seen for the Ar atom and all basis sets. Such behavior has not been observed for nuclear shielding of lighter atoms, as evidenced, for example, by the example of the PN molecule [15]. In this work, we have demonstrated that the scattering convergence of the aug-cc-pV*X*Z shieldings holds for the entire third row. Based on our results, we therefore propose to use the core-valence basis set families or the Jensen segmented contracted basis sets for calculations of shieldings of these nuclei, which could warrant the CBS estimations of NMR parameters shieldings being more reliable than results obtained using standard Dunning basis sets. The effect of electron correlation was relatively low (below 15%) for all the studied hydrides but increased significantly for systems with multiple bonds (PN, HSiCH). The incomplete results for H_3_PO, on the other hand, show quite good results of the HF method compared to CCSD(T). We can only speculate whether this is just a fortuitous error cancellation or an exception to the rule, and additional calculations on other molecules with multiple bonds are needed.

We also evaluated the effect of vibrational, temperature, and relativistic corrections to nuclear shieldings of the third-row elements considering them as additive factors to the equilibrium CBS values. For systems with single bonds, all corrections are rather small, being less than 4% of the CCSD(T)/CBS value estimated using the core-valence basis sets. The ZPVC and TC estimates were difficult for H_3_PO and HSiCH due to their high anharmonicity and/or method failure and different levels provided significantly different values. On the other hand, this was not observed for PN, where comparable ZPVCs were achieved regardless of what level of theory was used. Interestingly, we obtained the highest relativistic correction to nuclear shielding for phosphorus in PN. The correction was ~20% of the CCSD(T)/CBS value, while it was substantially lower (<7%) for other elements. Note that we estimated the four-component RC at the DFT level for cost/benefit reasons, which turned out to be insufficient for estimating the isotropic PN shielding. Therefore, RC estimates (calculated as the difference between relativistic and non-relativistic values) may also be affected by the inadequacy of the DFT theory. The complete CCSD(T) description may be a solution with correct RC calculations, but this is not available to us and, moreover, this method is too uneconomical for most common molecules.

As an extension of this study, the ^33^S NMR shieldings of 2-thiouracil were estimated as an example of a real medium-sized molecule. We employed two different approaches. One was based on the standard description of the system using the same basis set for all atoms, while the other employed the locally dense basis set approach [66,67,68,69]. For both approaches, we observed a scattering of ^33^S shieldings for the aug-cc-pV*X*Z basis set family comparable to H_2_S, while a smooth convergence is seen for aug-cc-pCV*X*Z. The change in estimated CBS values due to the LDBS approach is ~10% of the CBS value when all atoms were described using aug-cc-pV*X*Z or aug-cc-pCV*X*Z.

## 4. Materials and Methods

Most calculations were performed using the Gaussian 16 [79] and CFOUR-2.1 [80] programs. Zero-point vibrational corrections were calculated with the S4 program [81]. 

### 4.1. Geometry

Geometries of all compounds in this work were either taken from previous reports or optimized as described below. The previously reported optimized structure (CCSD(T)/aug-pc-4) of phosphorus mononitride [15] (PN) was used in this study, with an interatomic separation of 1.49466464 A°. Hydrogen sulfide (H_2_S) geometry parameters of SH = 1.3376 A° and HSH = 92.11 A° obtained by the infrared and microwave spectral analysis were taken from [57]. Phosphine (PH_3_) geometry (PH = 1.42002 A° and HPH = 93.3454 A°) was taken from [57] The X-ray structure of 2-thiouracil (2-TU) was taken from [64] and reoptimized at the B3LYP/aug-cc-pV5Z level to gain accurate C–H and N–H bond lengths. Geometries of all other model compounds (NaH, NaF, MgH_2_, AlH_3_, HsiCH, H_3_PO, and HCl) in this study were achieved by their optimization at the B3LYP/aug-cc-pV5Z level. In order to describe the geometry influence on the resulting NMR property, the B3LYP geometry of NaH was assessed against the CCSD(T) geometry and the experimental geometry ([56]; Na-H = 1.8874 A°).

### 4.2. NMR Shieldings

The following basis set families, acquired from EMSL [82,83,84,85] were used in the calculations of NMR shieldings: (aug)-cc-pV*X*Z, where X = D, T, Q, 5 and 6; (aug)-cc-pCV*X*Z, where X = D, T, Q and 5; aug-cc-pwCV*X*Z, where X = D, T, Q and 5; and Jensen aug-pcJ-*n*, and aug-pcSseg-*n*, where *n* = 1, 2, 3 and 4. In addition, the Karlsruhe-type all-electron relativistic split-valence (x2c-SVPall-s), triple-ζ (x2c-TZVPPall-s) and double-polarized quadruple-ζ (x2c-QZVPPall-s) basis sets for two-component calculations of NMR shieldings, as well as the Douglas–Kroll-type aug-cc-pV*X*Z-DK, were applied as indicated below and taken from EMSL [82,83,84,85].

The GIAO NMR parameters were calculated at the SCF-HF and CCSD(T) levels using the CFOUR program, and at the DFT-B3LYP level with Gaussian 16 [79]. The all-electron CCSD(T) nuclear shieldings were calculated with the CFOUR-2.1 program [80]. The locally dense basis sets (LDBS) approach [66,67,68,69] was applied for the B3LYP calculation of the ^33^S NMR shielding constants in 2-TU to reduce the computational time. Thus, C, H, N, O atoms were described by the 6-31G* basis set and the sulfur atom was calculated with the aug-cc-pV*X*Z or aug-cc-pCV*X*Z basis set families. In the case of hydrogen atom, aug-cc-pCV*X*Z = aug-cc-pV*X*Z. Thus, the aug-cc-pV*X*Z basis sets were used for hydrogen. Shielding constants, shielding anisotropies and individual shielding components were plotted against the basis set cardinal number *X*, or the number of basis functions (b.f.). The latter approach allowed a better discrimination between the size of different basis set series but essentially produced the same CBS value. Finally, the CBS values of calculated NMR parameters, Y(CBS), were estimated using the 2-parameter formula [86,87] (Equation (1)).

The GIAO NMR parameters were calculated at the SCF-HF and CCSD(T) levels using the CFOUR program, and at the DFT-B3LYP level with Gaussian 16 [79]. The all-electron CCSD(T) nuclear shieldings were calculated with the CFOUR-2.1 program [80]. The locally dense basis sets (LDBS) approach [66,67,68,69] was applied for the B3LYP calculation of the ^33^S NMR shielding constants in 2-TU to reduce the computational time. Thus, C, H, N, O atoms were described by the 6-31G* basis set and the sulfur atom was calculated with the aug-cc-pV*X*Z or aug-cc-pCV*X*Z basis set families. In the case of hydrogen atom, aug-cc-pCV*X*Z = aug-cc-pV*X*Z. Thus, the aug-cc-pV*X*Z basis sets were used for hydrogen. Shielding constants, shielding anisotropies and individual shielding components were plotted against the basis set cardinal number *X*, or the number of basis functions (b.f.). The latter approach allowed a better discrimination between the size of different basis set series but essentially produced the same CBS value. Finally, the CBS values of calculated NMR parameters, Y(CBS), were estimated using the 2-parameter formula [86,87] (Equation (1)).

(1) Y(X)=Y(CBS)+AX3

In this formula, Y(CBS) and A are the fitted parameters and X (or b.f.) is the cardinal number (or the number of basis functions) of the basis set. We used X=n+1 in the case of Jensen basis sets (i.e., pc-1 basis set corresponds to the double-ζ quality).

In the case of three systematically growing x2c- basis sets, we expected no regular convergence of energy and related parameters. However, to obtain some indication of trends in calculated nuclear shieldings, we also performed the 2-parameter fit. In this case, the fitting was performed for all three data points. Obviously, this was an empirical treatment of the data (produced by these basis sets). Despite such crude approximations, the obtained CBS-like values were often close to numbers obtained with the Dunning or Jensen-type basis sets.

### 4.3. Zero-Point Vibrational and Thermal Corrections

The nuclear potential of studied compounds was expanded to a Taylor series up to fourth powers of all normal-mode coordinates according to Equation (2) to estimate the ZPVC effect on calculated NMR shieldings [88].

(2) $$V=\frac{1}{2}\sum_{i=1}\omega_{i}^{2}Q_{i}^{2}+\frac{1}{6}\sum_{i=1}\sum_{j=1}\sum_{k=1}c_{\mathrm{ijk}}Q_{i}Q_{j}Q_{k}+\frac{1}{24}\sum_{i=1}\sum_{j=1}\sum_{k=1}\sum_{l=1}d_{\mathrm{ijkl}}Q_{i}Q_{j}Q_{k}Q_{l}$$

We considered only cubic ($c_{\mathrm{ijk}}$) and semi-diagonal quartic constants ($d_{\mathrm{ijkl}}$; with two or more identical indices), as a single numerical differentiation of harmonic force fields provides them. Isotropic nuclear magnetic shieldings were calculated for vibrational ground state $\psi_{n}$ as $\sigma_{n}=\psi_{n}{|\sigma |\psi }_{n}$, where $\sigma =\sigma_{0}+\sum_{i}\sigma_{1,i}Q_{i}+\frac{1}{2}\sum_{i,j}\sigma_{2,\mathrm{ij}}Q_{i}Q_{j}$. The $\sigma_{1}$ and $\sigma_{2}$ are the first and the second normal-mode isotropic shielding derivatives that were obtained numerically as described elsewhere [89]. The wave function is expanded in the harmonic oscillator basis within the second-order degeneracy-corrected perturbational (PT2) approach [90] providing thus the zero-point vibrational corrections (ZPVCs). All geometries were optimized at the B3LYP/aug-cc-pVQZ level. The anharmonic force field and the shielding derivatives were obtained at the same level of theory. Alternatively, we calculated the anharmonic force field (as we did the optimization) at the CCSD(T)/aug-pc-2 level and the shielding derivatives at the BHandHLYP/aug-pcSseg-4 level. The Hessian and NMR computations for displaced geometries (performed in normal modes) were carried out using the Gaussian 16, while the anharmonic vibrational averaging was executed using program S4. The temperature-corrected shieldings (TCS) were obtained as $\sigma =\sigma_{0}+0.25\sigma_{\mathrm{ii}}\exp\left(-\omega_{i}/\mathrm{kT}\right){\left[1-\exp\left(-\omega_{i}/\mathrm{kT}\right)\right]}^{-1}$. Then, the pure temperature correction was obtained as the TCS-ZPVC. Note that our simplified estimation of the TC does not include centrifugal distortion, which may represent a large contribution to the TC.

### 4.4. Relativistic Corrections

We employed the Respect 5.2.0 [91] code to obtain the relativistic corrections to theoretical GIAO NMR shielding constants. We compared the full four-component Dirac–Kohn–Sham shieldings [92,93], calculated with the B3LYP functional and the aug-cc-pVTZ basis set, with values achieved with one-component Kohn–Sham Hamiltonian at the same level. For PN, we examined the basis set dependence of relativistic corrections using the aug-cc-pV*X*Z series, where X = D, T, Q. Alternatively, the relativistic correction was achieved also at the KT2 level with the uncontracted pcS-3 basis set. The correction for Ar was obtained using the uncontracted Dyall aug-cvtz basis set. Moreover, since Ar is not defined as an NMR-active nucleus in by default, we obtained RC for Ar by interpolation of theoretical values of He, Ne, Kr, and Xe using the trendline, where Z is the atomic number of a nucleus.

## 5. Conclusions

A detailed test of HF-SCF, B3LYP and CCSD(T) of the apparent irregularity of the convergence of nuclear magnetic shielding tensors with respect to increasing the size of the aug-cc-pV*X*Z basis set has been performed for selected isolated molecules containing nuclides of the third row of the periodic table of elements. The scattered patterns of nuclear shieldings calculated by the three selected methods and using Dunning basis sets with a regularly increasing cardinal number *X* were observed for the studied compounds. In contrast to NMR shieldings, regular and exponential decays were observed for energies calculated using the same approach. The use of the aug-cc-pCV*X*Z core-valence basis set family or the segmented-contracted aug-cc-pcSseg-*n* basis sets (slightly smaller than the former one) improved the behavior of the calculated NMR shieldings with smooth convergence towards the CBS limits. In addition, the x2c-Def2 basis sets, being significantly smaller than the aug-cc-pCV*X*Z or aug-cc-pcSseg-*n* basis sets, provided results close to the CBS limit for the latter two families. Obtained results point to the necessity of using the aug-pcSseg-*n* or aug-cc-pCV*X*Z basis sets if one attempts to obtain converged (or close to saturation) nuclear shieldings of the third-row elements. As a cheaper alternative, the x2c-Def2 basis sets could also be employed for reliable prediction of nuclear shieldings for compounds containing elements from the third row of the periodic table.

Additionally, we estimated the effect of vibrational, temperature, and relativistic corrections on the predicted shieldings of the third-row elements. The vibrational corrections were estimated using the second-order degeneracy-corrected perturbational approach. The relativistic corrections were obtained at the full four-component Dirac–Kohn–Sham basis. We can conclude that all corrections are relatively small, amounting to less than 4% of the CCSD(T)/CBS value, for systems containing only single bonds. Estimates of the vibrational and temperature corrections were less reliable for H_3_PO and HSiCH due to the high anharmonicity of these molecules. Abnormally high relativistic corrections, reaching ~20% of the CCSD(T)/CBS value, were observed for phosphorus in PN, while the corrections were substantially lower (~7% of the CCSD(T)/CBS value) for other tested molecules.

## Figures and Tables

**Table 1:** List of applied basis sets and their abbreviations.

Basis Set/Full Name	Abbreviation
(aug)-cc-pV*X*Z	(a)*X*Z
(aug)-cc-pCV*X*Z	(a)C*X*Z
(aug)-cc-pwV*X*Z	(a)w*X*Z
(aug)-cc-pwCV*X*Z	(a)wC*X*Z
aug-pc-*n*	apc*n*
aug-pcSseg-*n*	apcSseg*n*
aug-pcJ-*n*	apcJ*n*
Karlsruhe-type basis set	x2c-Def
x2c-*S*VPall-s	x2c*S*V
x2c-*T*ZVPPall-s	x2c*T*Z
x2c-*Q*ZVPPall-s	x2c*Q*Z
Complete basis set limit	CBS
Zero-point vibration correction	ZPVC
Temperature correction	TC
Relativistic correction	RC
Gauge-including atomic orbital	GIAO
Polarized continuum model of solvent	PCM

**Table 2:** Calculated B3LYP/CBS ^a 31^P and ^15^N nuclear shielding components, isotropic shieldings and shielding anisotropy of PN ^b^ with respect to the number of basis functions (b.f.).

	^31^P				^15^N			
CBS Type	σ_xx_	σ_zz_	σ_iso_	σ_aniso_	σ_xx_	σ_zz_	σ_iso_	σ_aniso_
**aV*X*Z**								
(*5-6*)	−575.605	966.522	−61.563	1542.127	−829.620	341.858	−439.128	1171.478
**aCV*X*Z**								
(*Q-5*)	−572.407	966.332	−59.494	1538.739	−827.668	341.860	−437.825	1169.527
**awCV*X*Z**								
(*Q-5*)	−572.721	966.333	−59.703	1539.052	−828.236	341.860	−438.204	1170.095
**Apc*n***								
(*3-4*)	−572.069	966.686	−59.150	1538.755	−829.711	341.862	−439.186	1171.573
**apcSseg*n***								
(*3-4*)	−572.596	966.399	−59.598	1538.995	−828.249	341.870	−438.210	1170.119
**apcJ*n***								
(*3-4*)	−573.360	966.087	−60.210	1539.447	−828.154	341.853	−438.151	1170.007
Method	Literature							
CCSD(T)/aV*X*Z ^c^			58.080	1362.090				
CCSD(T)/aCV*X*Z ^c^			59.090	1361.250				
B3LYP/6-311++G** ^d^			−57.48				−406.54	
CCSD(T)/15s12p4d3f2g ^e^			49.0					

^a^ Basis sets selected for fitting are in parenthesis (e.g., CBS(*5-6*) is calculated using the Dunning basis sets aV*5*Z and aV*6*Z; for Jensen basis sets, CBS(*3-4*) denotes extrapolation with apc*3* and apc*4*). ^b^ All NMR calculations were performed using the CCSD(T)/aug-pc-4 geometry (1.49466464 A°). ^c^ From [15]. ^d^ From [54], where the authors also cited the experimental value of 53.0 ppm. ^e^ From [55].

**Table 3:** Calculated CBS nuclear shielding values (in ppm) for studied species ^a,b^.

Methods	HF-SCF	B3LYP	CCSD(T)	Δ (%) from CCSD(T)	
				SCF	B3LYP
**NaH**					
aV*X*Z(T-5)	562.384	565.305	549.057	2.4	3.0
aCV*X*Z(T-5)	565.269	572.698	569.555	−0.8	0.6
apcSseg-*n*(2-4)	565.478	572.789	572.180	−1.2	0.1
**MgH_2_**					
aV*X*Z(T-5)	475.048	397.813	441.802	7.5	−10.0
aCV*X*Z(T-5)	462.948	426.089	447.156	3.5	−4.7
apcSseg-*n*(2-4)	460.706	426.588	443.87	3.8	−3.9
**AlH_3_**					
aV*X*Z(Q-6)	340.417	260.370	301.061	13.1	−13.5
aCV*X*Z(T-5)	346.671	267.211	307.762	12.6	−13.2
apcSseg-*n*(2-4)	344.362	265.630	305.775	12.7	−13.1
**SiH_4_**					
aV*X*Z(T-5)	489.275	445.37	483.294	1.2	−7.8
aCV*X*Z(T-5)	477.703	435.416	470.854	1.5	−7.5
apcSseg-*n*(2-4)	473.790	434.990	468.972	1.0	−7.2
HSi≡CH					
aV*X*Z(T-5)	917.646	498.605	619.338	48.2	−19.5
aCV*X*Z(T-5)	907.666	501.293	630.101	44.1	−20.4
apcSseg-*n*(2-4)	915.536	501.697	628.762	45.6	−20.2
**PH_3_**					
aV*X*Z(Q-6)	576.501	553.876	596.957	−3.4	−7.2
aCV*X*Z(T-5)	581.367	557.847	603.326	−3.6	−7.5
apcSseg-*n*(2-4)	580.892	557.661	588.578	−1.3	−5.3
**H_3_PO**					
aV*X*Z(5-6)	398.514	349.201	-	-	-
aCV*X*Z(T-5)	397.276	346.109	-	-	-
apcSseg-*n*(2-4)	396.549	347.681	-	-	-
**PN ^a^**					
aV*X*Z(5-6)	−91.460	−58.882	58.080	−257.5	−201.4
aCV*X*Z(5-6)	−91.560	−60.030	59.090	−255.0	−201.6
apcSseg-*n*(3-4)	−90.720	−58.833	58.780	−254.3	−200.1
**H_2_S**					
aV*X*Z(Q-6)	708.776	694.933	736.852	−3.8	−5.7
aCV*X*Z(T-5)	712.644	698.246	741.209	−3.9	−5.8
apcSseg-*n*(2-4)	715.929	698.071	742.245	−3.5	−6.0
**HCl**					
aV*X*Z(Q-6)	944.476	930.256	955.745	−1.2	−2.7
aCV*X*Z(T-5)	931.858	946.403	957.943	−2.7	−1.2
apcSseg-*n*(2-4)	946.06	931.705	957.3498	−1.2	−2.7
**Ar**					
aV*X*Z(Q-6)	1237.659	1238.172	1237.509	0.0	0.1
aCV*X*Z(T-5)	1237.660	1237.868	1237.924	0.0	0.0
apcSseg-*n*(2-4)	1237.534	1237.930	1237.516	0.0	0.0

^a^ Results of this work and partially from [15]. ^b^ CBS(*5-6*) denotes Dunning-type basis set extrapolation using V*5*Z and aV*6*Z. CBS(*2-4*) obtained with Jensen basis sets is constructed with apc*2*, apc*3* and apc*4.*

**Table 4:** Zero-point vibrational, thermal, and relativistic corrections to NMR shieldings of the third-row elements (in ppm).

	NaH	MgH_2_	AlH_3_	SiH_4_	HSi≡CH	PH_3_	H_3_PO	PN	H_2_S	HCl	Ar
**This work**											
Isotropic σ	569.56	447.16	307.76	470.85	628.76	603.33	389.34 *	59.09	741.21	957.94	1237.92
ZPVC^mixed^	1.03	6.61	−0.13	1.85	30.77	−5.60	69.39	−4.40	−21.12	−17.35	-
ZPVC^B3LYP^	0.29	10.66	−1.06	20.28	−0.69	−10.81	−4.72	−6.21	−22.36	−18.29	-
TC (273K)	−0.01	1.36	0.01	−0.75	−1.30	−0.42	−0.15	−0.04	−0.51	−0.42	-
RC^KT2^	8.08	9.96	11.52	14.87	20.99	20.80	18.31	11.45	27.26	33.21	33.72
RC^B3LYP^	7.74	9.97	11.97	14.92	25.84	18.86	22.35	12.37^#^	24.80	32.16	-
Final value	577.59	467.79	278.12	506.05	653.91	611.38	406.97	65.25	743.65	971.81	1271.64
**Literature**											
Isotropic σ				470.64 ^a^		605.83 ^a^			737.92 ^a^	961.92 ^f^	1237.50 ^e^; 1237.76 ^g^
ZPVC				−1.41 ^a^		−9.50 ^c^			−20.86 ^a^	−17.09 ^f^	
TC (273K)				0.01 ^a^		−0.32 ^a^			−0.89 ^a^	−0.59 ^f^	
RC										31.82 ^f^	37.52 ^e^
Final value				469.24		596.01			716.17	976.06	1275.02; 1275.28; 1273.89 ^h^
Exp. Total						594.45 ^d^			717.24 ^a^	-	
Exp. σ				475.3 ± 10 ^b^		−266.10 ^e^					

Isotropic values—CCSD(T)/CBS results from the 2-parameter fit of the aug-cc-pCVXZ values. See Table 3 (* for H3PO—the CCSD(T)/aug-cc-pCVQZ value); PVC^mixed^—CCSD(T)/aug-pc-2 //BHandHLYP/aug-pcSseg-4; ZPVC^B3LYP^—B3LYP/aug-cc-pVQZ; TC (at 273K)—B3LYP /aug-cc-pVQZ; RC^KT2—^KT2/pcS-3; RC^B3LYP^—B3LYP/aug-cc-pVTZ (for PN—the B3LYP /aug-cc-pVQZ value). Final value (this work) = isotropic σ + ZPVC^B3LYP^ + TC + RC^B3LYP^. ^a^ [57] ^b^ [75]; ^c^ [55]; ^d^ [76]; ^e^ [77]; ^f^ [72]; ^g^ [78]; ^h^ [73].

## Data Availability

All data are available in the text and the Supplementary Material.

## Supplementary Materials

The following supporting information can be downloaded at: https://www.mdpi.com/article/10.3390/molecules27238230/s1: Additional figures and tables showing convergence patterns of nuclear shieldings of third-row elements in test compounds, calculated with the SCF-HF, B3LYP-DFT, and CCSD(T) methods, combined with the aug-cc-pV*X*Z, aug-cc-pCV*X*Z, aug-pcSseg-*n* and x2c-Def2 basis set series. The calculated energies are also included.
