# Supplementary material for: Electron Correlation or Basis Set Quality: How to Obtain Converged and Accurate NMR Shieldings for the Third-Row Elements?
Source: Molecules. 2022 Nov 25;27(23):8230. doi: 10.3390/molecules27238230 (PMC9737175; doi:10.3390/molecules27238230)
Supplement: Supplementary file 1 [file molecules-27-08230-s001.zip › molecules-2040830-supplementary.pdf]

molecules MDPI

Supplementary Materials

Electron Correlation or Basis Set Quality: How to Obtain Converged and Accurate NMR Shieldings for the Third-Row Elements?

Kacper Rzepiela [1], Jakub Kaminský [2,*], Aneta Buczek [1], Małgorzata A. Broda [1] and Teobald Kupka [1,*]

[1] Faculty of Chemistry, University of Opole, 48 Oleska Street, 46-052 Opole, Poland [2] Institute of Organic Chemistry and Biochemistry of the CAS, Flemingovo nám. 2, 166 10 Prague, Czech Republic

* Correspondence: kaminsky@uochb.cas.cz (J.K.); teobaldk@gmail.com (T.K.)

Supplemental Material

S1.1 Sensitivity of ^{23}Na parameters to the basis set quality

The ^{23}Na nuclear shielding values calculated at the B3LYP/aug-cc-pV*X*Z and B3LYP/aug-cc-pCV*X*Z levels for NaH, but also for NaF are gathered in Table S6A. Table also presents NMR shieldings for NaF and NaH calculated at the HF-SCF and CCSD(T) levels with same basis sets as for B3LYP. Similarly, Table S6B then summarizes energies for two selected sodium species calculated at the B3LYP, HF-SCF and CCSD(T) levels with the aug-cc-pV*X*Z and aug-cc-pCV*X*Z basis sets.

The convergence patterns of ^{23}Na isotropic shieldings according to the number of basis functions for NaH calculated at the HF-SCF, B3LYP-DFT and CCSD(T) levels of theory, combined with the aug-cc-pV*X*Z, aug-cc-pCV*X*Z, aug-pcSseg-*n* and x2c-Def2 basis set families are shown in Figure S7. It is apparent that ^{23}Na isotropic shieldings predicted with the aug-cc-pV*X*Z series produce scattered results and a smooth convergence is for shieldings produced with the aug-pcSseg-*n* family. Interestingly, despite their small size, the x2c-Def2 basis set family performs fairly accurate in comparison to Jensen's or core-valence basis sets. Thus, apart from aug-cc-pCV*X*Z basis sets, the x2c-Def2 basis sets could be recommended for shielding calculations of ^{23}Na nuclei. In addition, Figure S8 depicts very similar ^{23}Na shielding patterns for NaF as for NaH with respect to the cardinal number *X* and the number of basis functions calculated with the B3LYP functional and aug-cc-pV*X*Z and aug-cc-pCV*X*Z basis set families. Note that the B3LYP/aug-cc-pV*X*Z and B3LYP/aug-cc-pCV*X*Z calculated energies of NaH and NaF show typical (exponential) patterns as seen in Figure S9.

According to Table S6A, the CCSD(T)/CBS (estimated with the core-valence basis sets) ^{23}Na isotropic nuclear shielding in NaH is 569.555 ppm. When we consider also ZPVC of 0.29 ppm,

RC of 7.74 ppm, and TC of -0.01 ppm (Table 4), the total shielding changes to its final value of 577.585 ppm.

S1.2 Sensitivity of ^{25}Mg parameters to the basis set quality

The ^{25}Mg nuclear shielding values calculated at the B3LYP level with the aug-cc-pV*X*Z, aug-cc-pCV*X*Z, aug-pcSseg-*n,* and x2c-Def2 basis sets for MgH_2 are gathered in Table S7A, as well as the estimated CBS values. Analogously, Table S6B summarizes electronic energies of MgH_2 calculated at the same levels for the first two basis set families. Figure S10 then, for clearer picture of the magnesium shielding behavior with increasing basis set, depicts convergence patterns of ^{25}Mg NMR shieldings for MgH_2 calculated with the CCSD(T) method. Again, four basis set families, aug-cc-pV*X*Z, aug-cc-pCV*X*Z, aug-pcSseg-*n*, and x2c-Def2 were used. Similarly to NaH, the results obtained with the aug-cc-pV*X*Z basis sets exhibit a slightly scattered and not converging behavior with increasing the basis set size. Interestingly, the difference between isotropic shieldings calculated with the lowest and the highest basis set (aug-cc-pVDZ and aug-cc-pV5Z) are -36, -98, and -73 ppm for HF-SCF, B3LYP and CCSD(T), respectively (see Table 7A). Besides, the difference between the aug-cc-pCV5Z and aug-cc-pV5Z ^{25}Mg shielding calculated with these three methods was 7, -35 and -22 ppm, respectively. As expected, the results obtained with the core-valence basis set family, as well as with Jensen's and Karlsruhe type series smoothly converge towards the CBS limit (Figure S10).

According to Table S7A, the CCSD(T)/CBS (estimated with the core-valence basis sets) ^{25}Mg isotropic nuclear shielding in MgH_2 is 447.156 ppm. When we consider also ZPVC of 10.66 ppm, RC of 9.97 ppm, and TC of 1.36 ppm (Table 4), the total shielding changes to its final value of 467.786 ppm.

In contrast, we can see a smooth energy convergence for MgH_2 calculated with the B3LYP functional and the two basis sets in Figure S10B.

S1.3 Sensitivity of ^{27}Al parameters to the basis set quality

As in previous sections, convergence patterns of ^{27}Al NMR shielding constants for AlH_3, calculated with the HF-SCF, B3LYP and CCSD(T) methods, combined with the aug-cc-pV*X*Z, aug-cc-pCV*X*Z, aug-pcSseg-*n*, and x2c-Def2 basis sets are gathered in Table S8A and the coupled cluster results are shown graphically in Figure S11A. Again, the CCSD(T) results obtained with the aug-cc-pV*X*Z basis sets show an irregular zig-zag pattern, which doesn't allow any reliable extrapolation of shieldings to the CBS limit. This is solved by utilizing the core-valence or Jensen's basis set family that converge smoothly towards the CBS following an exponential decay curve. The results, calculated with Karlsruhe basis set series are also near the CBS limit. Observed scatter of ^{27}Al isotropic shieldings calculated at CCSD(T)/aug-cc-pV*X*Z level of theory is significant (ca. -74, -7, -15 ppm for $X = T \rightarrow D$, $Q \rightarrow T$ and $5 \rightarrow Q$). To extend the choice of core-valence basis sets we also tested the performance of HF-SCF and CCSD(T) calculations with the aug-cc-pwV*X*Z family. The CCSD(T)/CBS (estimated with the aug-cc-pwV*X*Z basis sets) ^{27}Al isotropic nuclear shielding in AlH_3 was estimated as 307.762 ppm changing to 318.672 ppm after consideration of ZPVC, RC, and TC (-1.06 ppm, 11.92 ppm, and 0.01 ppm; see Table 4).

Table S8B also adds information of the B3LYP and CCSD(T) calculated energies for AlH_3 using aug-cc-pV*X*Z and aug-cc-pCV*X*Z series of basis sets and Figure S11B shows the corresponding regular convergence of the B3LYP/aug-cc-pV*X*Z and B3LYP/aug-cc-pCV*X*Z calculated energy of AlH_3.

S1.4 Sensitivity of ^{29}Si parameters to the basis set quality

Another NMR nucleus in the third row is ^{29}Si. Convergence patterns of ^{29}Si NMR shielding constants for SiH_4 calculated again with the HF-SCF, B3LYP and CCSD(T) methods, combined with the aug-cc-pV*X*Z, aug-cc-pCV*X*Z, aug-pcSseg-*n*, and x2c-Def2 basis sets are gathered in Table S9A and are depicted in Figure S12A. As seen before, the aug-cc-pV*X*Z basis sets provide again an irregular pattern that is impossible to reliably extrapolate to the CBS limit. However, the core-valence basis set family gives shieldings smoothly converging towards CBS. In case of SiH_4, the change of isotropic shieldings upon increasing the basis set size observed for aug-cc-pV*X*Z (-67, 14, -34 ppm for X = T$\rightarrow$D, Q$\rightarrow$T and 5$\rightarrow$Q) is comparable to changes of the ^{27}Al NMR parameters. The estimated CCSD(T)/CBS(aug-cc-pCV*X*Z) of ^{29}Si isotropic nuclear shielding in SiH_4 is 470.854 ppm. When ZPVC, RC, and TC (20.28 ppm, 14.92 ppm, and -0.75 ppm; Table 4) are included the final value increases to 506.054 ppm. As before, the Jensen's and Karlsruhe type basis sets produce regularly converging patterns of ^{29}Si isotropic nuclear shielding.

Table S9B compares the B3LYP and CCSD(T) energies of SiH_4 calculated with the aug-cc-pV*X*Z and aug-cc-pCV*X*Z basis sets. In Figure S12B we can see regular energy convergences for SiH_4 calculated using the B3LYP functional and the two Dunning's basis sets.

As an example of a silicon-containing molecule with the triple bond we picked $HSi\equiv CH$. Its NMR shieldings were calculated again with the HF-SCF, B3LYP, and CCSD(T) methods, combined with the aug-cc-pV*X*Z, aug-cc-pCV*X*Z, aug-pcSseg-*n*, and x2c-Def2 basis sets. All corresponding silicon nuclear shieldings are gathered in Table S9C. Since $HSi\equiv CH$ contains a triple bond between silicon and carbon, it represents an interesting model because it is supposed

to be highly anharmonic and ZPVC may play a significant role. This appeared partially true, as seen in Table 4. However, the calculated ZPVC varies with the level of theory (30.77 ppm for the mixed CCSD(T)/BHandHLYP level and -0.69 ppm for B3LYP). Moreover, due to its nature, the standard perturbational approach fails when the lowest vibrational modes were included in the PT2 formula. Thus, the contribution of the three lowest modes had to be excluded from the ZPVC estimates. Nevertheless, ZPVC for $HSi \equiv CH$ may be unreliable and deserves further study in the future. Also, electron correlation is substantial (about 151 ppm on ^{29}Si) as documented in Table S9C.

S1.5 Sensitivity of ^{33}S parameters to the basis set quality

Another NMR active nucleus of the third row lying in the sixth group is ^{33}S. All ^{33}S nuclear shielding values are gathered in Table S10A for H_2S and are calculated using the HF-SCF, B3LYP and CCSD(T) methods combined with the aug-cc-pV*X*Z, aug-cc-pCV*X*Z, aug-pcSseg-*n*, and x2c-Def2 basis sets. In Table S10B there are the corresponding B3LYP and CCSD(T) energies calculated with the aug-cc-pVXZ and aug-cc-pCVXZ basis sets. Obviously, the nuclear shielding results obtained with the aug-cc-pV*X*Z basis set series are also scattered as we have seen for nuclei in other groups (Figure S13A). Thus, it is difficult to extrapolate these results to the CBS limit. However, the results obtained with the core-valence basis set family again converge towards CBS (see also ref.[12]) for X = T to 5. In case of H_2S, the magnitude of the scatter of isotropic shieldings calculated with CCSD(T)/aug-cc-pV*X*Z method is comparable to the ^{31}P NMR parameters (see changes of -29, -3, -22 and -1 ppm for X = T$\rightarrow$D, Q$\rightarrow$T, 5$\rightarrow$Q and 6$\rightarrow$5). The isotropic CCSD(T)/CBS(aug-cc-pCV*X*Z) ^{33}S nuclear shielding of 741.209 ppm from

Table S10A was corrected using ZPVC, RC, and TC (-22.36 ppm, 24.80 ppm, and -0.51 ppm; Table 4) and the final value of 743.649 ppm was obtained.

As for other elements, Figure S13B in Supporting Information reveals smooth convergences of H_2S energies calculated at the B3LYP level with the two basis sets.

S1.6 Sensitivity of ^{35}Cl parameters to the basis set quality

Nuclear shielding values calculated using HF-SCF, B3LYP and CCSD(T) methods combined with the aug-cc-pV*X*Z, aug-cc-pCV*X*Z, aug-pcSseg-*n*, and x2c-Def2 basis sets for the 3rd row halogen, chlorine, are gathered in Table S11A. The B3LYP and CCSD(T) calculated energies using the aug-cc-pV*X*Z, aug-cc-pCV*X*Z basis sets for HCl can be found in Table S11B. Figure S14A shows convergence patterns of ^{35}Cl NMR shielding constants for HCl calculated with the four selected basis sets and using the CCSD(T) method. As before, the aug-cc-pV*X*Z basis set family produces unreliable and scattered results upon increasing the basis set size. Similarly as for previous nuclei, it is difficult to extrapolate these results to the CBS limit according to the X = T, Q, 5 and 6 data. On the other hand, corresponding points obtained with the aug-cc-pCV*X*Z show a nice decaying convergence pattern for X = T to 5. In case of HCl, the scatter of isotropic shieldings calculated with aug-cc-pV*X*Z is significantly smaller than for ^{33}S NMR parameters (see changes of about -16, -2, -11 and -1 ppm for X = T$\rightarrow$D, Q$\rightarrow$T, 5$\rightarrow$Q and 6$\rightarrow$5). The CBS (CCSD(T)/aug-cc-pCV*X*Z) ^{35}Cl isotropic nuclear shielding in HCl was estimated as 957.943 ppm, which was later corrected by ZPVC (-18.29 ppm), RC (32.16 ppm), and TC (-0.42 ppm) to its final value of 971.813 ppm (see Table 4). There is also a nice convergence of chlorine shieldings calculated with the aug-pcSseg-*n* basis set family. The x2c-Def2 basis sets produce results close to the CBS value, estimated for the aug-cc-pCVXZ basis set hierarchy.

Theoretical smooth energy convergences for HCl calculated with the B3LYP functional and the two Dunning's type basis sets are shown in Figure S14B in Supplemental Material.

S1.7 Sensitivity of ^{39}Ar parameters to the basis set quality

Convergence patterns of hypothetical ^{39}Ar NMR shielding constants calculated at the CCSD(T) level using the aug-cc-pV*XZ*, aug-cc-pCV*XZ*, aug-pcSseg-*n*, and x2c-Def2 basis sets are shown in Figure S15A and the individual HF-SCF, B3LYP, and CCSD(T) shieldings, as well as the estimated CBS values, are gathered in Table S12A. In this case, the results obtained with the aug-cc-pV*XZ* basis sets show a fairly regular convergence pattern. In contrast to previous hydrides, the ^{39}Ar shieldings are nearly saturated and converge is starting from smaller basis sets (compare results obtained with aug-cc-pCVTZ, aug-cc-pCVQZ and aug-cc-pCV5Z). Thus, scattering of ^{39}Ar isotropic shieldings calculated at the B3LYP/aug-cc-pV*XZ* level is significantly smaller (0.18, 0.01, 0.14, and 0.13 ppm for X = T→D, Q→T, 5→Q, and 6→5, resp.) than that, which is observed for the ^{35}Cl NMR parameters. Note that the estimated CBS (CCSD(T)/aug-cc-pCV*XZ*) value of the ^{39}Ar isotropic nuclear shielding is 1237.924 ppm, so the observed changes with increasing basis set size are negligible. Besides, the aug-pcSseg-*n* and aug-cc-pV*XZ* basis sets perform similarly and their CBS results are about 0.5 ppm lower than the converged values for aug-cc-pCV*XZ*. On the other hand, shieldings calculated using Karlsruhe basis sets scattered more than expected. When the relativistic correction of 33.72 ppm is considered, we obtain the best value of 1271.644 ppm.

Figure S15B clearly reveals a smooth energy convergence for the argon atom calculated with the B3LYP functional and the two Dunning's basis sets.

Table S1A. Theoretical ^{31}P nuclear shielding values (in ppm) of PH_3 calculated at the B3LYP, HF-SCF, and CCSD(T) level using various basis sets.

**PH_3**				
**Basis set**	**b. f.**	**B3LYP**	**HF-SCF**	**CCSD(T)**
**aV*X*Z**				
D	54	629.029	645.500	669.295
T	119	577.881	602.124	625.354
Q	222	583.120	610.049	630.431
5	371	557.361	580.829	601.383
6	574	557.782	580.718	601.079
**CBS(Q-6)**		**553.876**	**576.501**	**596.957**
**aCV*X*Z**				
D	63	591.385	612.462	639.239
T	144	561.534	584.555	608.292
Q	272	558.319	581.774	604.194
5	457	558.027	581.523	603.368
**CBS(T-5)**		**557.847**	**581.367**	**603.326**
**apcSseg*n***				
0	26	618.515	665.870	678.359
1	60	571.99	597.015	621.910
2	128	559.65	582.699	600.605
3	248	557.74	581.030	602.087
4	414	557.89	581.042	602.033
**CBS(2-4)**		**557.661**	**580.892**	**602.184**
**x2c-*X*ZVPPall-s**				
x2c-SVPall-s	45	551.927	585.077	601.819
x2c-TZVPPall-s	96	558.755	583.521	604.065
x2c-QZVPPall-s	172	553.668	577.512	598.945
**CBS(1-3)**		**556.340**	**580.073**	**601.348**

Table S1B. The B3LYP and CCSD(T) energies (in a.u.) of PH_3

**PH_3**		
**Basis set**	**B3LYP**	**CCSD(T)**
**aV*X*Z**		
D	-343.16263598	-342.65674477
T	-343.18054477	-342.74522177
Q	-343.18524453	-342.76498669
5	-343.18943646	-342.89034025
6	-343.18998064	-342.96350363
**CBS(Q-6)**	**-343.19246832**	**-343.04039416**
**aCV*X*Z**		
D	-343.16593385	-342.860511137
T	-343.18569238	-343.026773502
Q	-343.19001447	-343.093932239
5	-343.19061671	-343.121495928
**CBS(T-5)**	**-343.19234535**	**-343.146144750**

Table S2A. The B3LYP ^{31}P and ^{15}N nuclear shielding components, isotropic shieldings and shielding anisotropy of PN[a]

**Basis set**	**b. f.**[b]	**^{31}P**				**^{15}N**			
		**σ_{xx}**	**σ_{zz}**	**σ_{iso}**	**σ_{aniso}**	**σ_{xx}**	**σ_{zz}**	**σ_{iso}**	**σ_{aniso}**
**aV*X*Z**									
aVDZ	50	-414.054	966.072	45.988	1380.126	-709.558	341.805	-359.104	1051.363
aVTZ	96	-525.830	965.611	-28.683	1491.441	-789.443	341.523	-412.455	1130.966
aVQZ	164	-472.400	965.436	6.879	1437.835	-813.679	341.814	-428.514	1155.493
aV5Z	258	-581.863	966.515	-65.737	1548.378	-824.679	341.831	-435.843	1166.510
aV6Z	382	-577.533	966.520	-62.849	1544.053	-828.098	341.849	-438.116	1169.948
**CBS(5-6)**		**-575.605**	**966.522**	**-61.563**	**1542.127**				
**CBS(Q-6)**						**-829.013**	**341.84628**	**-438.727**	**1170.860**
**aCV*X*Z**									
aCVDZ	63	-537.347	966.292	-36.134	1503.638	-742.096	341.882	-380.770	1083.977
aCVTZ	134	-569.885	966.146	-57.875	1536.031	-807.266	341.856	-424.226	1149.122
aCVQZ	243	-571.027	966.329	-58.575	1537.356	-820.945	341.851	-433.346	1162.796
aCV5Z	398	-572.093	966.331	-59.285	1538.424	-826.138	341.858	-436.806	1167.995
**CBS(T-5)**		**-571.792**	**966.350**	**-59.078**	**1538.142**	**-825.545**	**341.855**	**-436.411**	**1167.3988**
**awCV*X*Z**									
awCVDZ	63	-539.032	966.100	-37.322	1505.132	-745.086	341.738	-382.811	1086.824

awCVTZ	134	-568.374	966.094	-56.885	1534.468	-801.810	341.817	-420.601	1143.627
awCVQZ	243	-569.984	966.325	-57.881	1536.309	-818.980	341.850	-432.037	1160.830
awCV5Z	398	-572.098	966.331	-59.288	1538.428	-826.129	341.857	-436.800	1167.987
**CBS(T-5)**		**-571.430**	**966.354**	**-58.835**	**1537.783**	**-825.133**	**341.858**	**-436.136**	**1166.991**
**apc*n***									
apc-1	50	-516.962	966.843	-22.360	1483.806	-744.540	342.014	-382.355	1086.553
apc-2	96	-561.408	966.691	-52.042	1528.099	-814.133	341.964	-428.767	1156.097
apc-3	178	-568.151	966.688	-56.538	1534.839	-826.000	341.860	-436.714	1167.860
apc-4	286	-571.124	966.687	-58.520	1537.811	-828.816	341.862	-438.590	1170.678
**CBS(2-4)**		**-570.606**	**966.687**	**-58.175**	**1537.293**	**-828.886**	**341.851**	**-438.641**	**1170.736**
**apcSseg*n***									
apcSseg-1	59	-578.815	966.522	-63.702	1545.337	-808.622	342.061	-425.061	1150.683
apcSseg-2	111	-579.105	966.435	-63.925	1545.540	-827.185	342.022	-437.449	1169.206
apcSseg-3	198	-574.961	966.428	-61.165	1541.389	-828.395	341.851	-438.313	1170.246
apcSseg-4	305	-573.244	966.407	-60.027	1539.650	-828.289	341.865	-438.238	1170.154
**CBS(2-4)**		**-573.422**	**966.414**	**-60.143**	**1539.835**	**-828.475**	**341.839**	**-438.371**	**1170.314**
**apcJ*n***									
apcJ-1	76	-619.637	928.452	-103.607	1548.088	-	341.963	-427.553	1154.275

						812.312			
apcJ-2	137	-592.272	954.892	-76.551	1547.164	-828.234	341.864	-438.202	1170.098
apcJ-3	220	-583.702	958.924	-69.493	1542.625	-828.163	341.840	-438.162	1170.002
apcJ-4	332	-576.369	964.003	-62.911	1540.372	-828.156	341.849	-438.154	1170.006
**CBS(2-4)**		**-577.405**	**962.963**	**-63.948**	**1540.368**	**-828.146**	**341.842**	**-438.150**	**1169.988**

[a]All NMR calculations were performed at CCSD(T)/aug-pc-4 geometry (1.49466464 Å); [b]Number of basis functions

Table S2B. Diamagnetic (DSO) and paramagnetic (PSO) contributions (in ppm) to the phosphorus nuclear shielding of PN calculated at the B3LYP level using various basis sets.

PN				
	**aVDZ**	**aVTZ**	**aVQZ**	**aV5Z**
DSO	971.43	971.94	949.46	962.39
PSO	-925.45	-1000.60	-942.54	-1028.41
Total	45.98	-28.66	6.92	-66.02
	**aCVDZ**	**aCVTZ**	**aCVQZ**	**aCV5Z**
DSO	974.16	964.91	966.54	967.84
PSO	-1010.31	-1022.79	-1025.12	-1027.13
Total	-36.15	-57.88	-58.58	-59.29
	**apcSseg-1**	**apcSseg-2**	**apcSseg-3**	**apcSseg-4**
DSO	967.90	966.65	967.93	969.23
PSO	-1031.61	-1030.59	-1029.10	-1029.26
Total	-63.71	-63.94	-61.17	-60.03
	**apc-1**	**apc-2**	**apc-3**	**apc-4**
DSO	967.67	966.74	969.19	972.15
PSO	-990.04	-1018.77	-1025.72	-1030.59
Total	-22.37	-52.03	-56.52	-58.43
	**apcJ-1**	**apcJ-2**	**apcJ-3**	**apcJ-4**
DSO	933.19	956.84	959.13	959.04
PSO	-1036.80	-1033.42	-1028.64	-1021.98
Total	-103.62	-76.57	-69.51	-62.95

Table S3. The B3LYP ^{31}P and ^{15}N NMR parameters shown as differences between 2 cardinal numbers in PN (ppm)

Basis set	^{31}P				^{15}N			
	σ_{xx}	σ_{zz}	σ_{iso}	σ_{aniso}	σ_{xx}	σ_{zz}	σ_{iso}	σ_{aniso}
**aV*X*Z**								
(T→D)	-111.776	-0.461	-74.671	111.315	-79.885	-0.282	-53.350	79.603
(Q→T)	53.430	-0.175	35.562	-53.606	-24.236	0.291	-16.060	24.527
(5→Q)	-109.464	1.079	-72.616	110.543	-11.001	0.016	-7.328	11.017
(6→5)	4.330	0.006	2.889	-4.325	-3.419	0.019	-2.273	3.438
**aCV*X*Z**								
(T→D)	-32.539	-0.146	-21.741	32.392	-65.171	-0.026	-43.456	65.145
(Q→T)	-1.142	0.183	-0.700	1.325	-13.678	-0.005	-9.120	13.673
(5→Q)	-1.066	0.002	-0.710	1.068	-5.193	0.007	-3.460	5.200
**awCV*X*Z**								
(T→D)	-29.342	-0.006	-19.563	29.336	-56.725	0.079	-37.790	56.803
(Q→T)	-1.610	0.231	-0.996	1.841	-17.170	0.033	-11.436	17.203
(5→Q)	-2.113	0.006	-1.407	2.119	-7.149	0.007	-4.764	7.157
**apc*n***								
(2-1)	-44.446	-0.153	-29.681	44.293	-69.593	-0.050	-46.412	69.543
(3-2)	-6.743	-0.003	-4.496	6.740	-11.867	-0.104	-7.946	11.763
(4-3)	-2.973	-0.001	-1.983	2.972	-2.816	0.002	-1.877	2.818
**apcSsegn**								
(2→1)	-0.290	-0.087	-0.223	0.203	-18.563	-0.039	-12.388	18.524
(3→2)	4.143	-0.007	2.760	-4.150	-1.210	-0.170	-0.864	1.040
(4→3)	1.718	-0.021	1.138	-1.739	0.106	0.013	0.075	-0.092
**apcJn**								
(2→1)	27.365	26.441	27.057	-0.924	-15.923	-0.100	-10.649	15.823

$(3\rightarrow2)$	8.570	4.031	7.057	-4.539	0.072	-0.024	0.040	-0.095
$(4\rightarrow3)$	7.333	5.080	6.582	-2.253	0.006	0.010	0.008	0.003

TABLE S4. Calculated B3LYP/CBS[a] ^{31}P nuclear shielding components, isotropic shieldings and shielding anisotropy of PN[b] with respect to cardinal number (*X*) The difference (in %) between the CBS values estimated with respect to the cardinal number *X* and the number of basis functions b.f. is shown as Δ (%)

PN				
**Basis set**	σ_{xx}	σ_{zz}	σ_{iso}	σ_{aniso}
**aV*X*Z**				
X (5-6)	-571.585	966.527	-58.882	1538.112
Δ (%)	0.698	0.000	4.355	0.260
**aCV*X*Z**				
X (Q-5)	-573.211	966.333	-60.030	1539.545
Δ (%)	-0.141	0.000	-0.900	-0.052
**awCV*X*Z**				
X (Q-5)	-574.316	966.337	-60.764	1540.651
Δ (%)	-0.279	0.000	-1.778	-0.104
**apc*n***				
X (3-4)	-574.244	966.685	-60.600	1540.929
Δ (%)	-0.380	0.000	-2.452	-0.141
**apcSseg*n***				
X (3-4)	-571.441	966.385	-58.833	1537.826
Δ (%)	0.202	0.001	1.284	0.076
**apcJ*n***				
X (3-4)	-568.675	969.332	-56.005	1538.008
Δ (%)	0.817	-0.336	6.983	0.093
CCSD(T)/aV*X*Z[c]			58.080	1362.090
CCSD(T)/aCV*X*Z[c]			59.090	1361.250
**Literature**				
PBE1PBE/6-311G(2d,2p)[d]			35.7	
B3LYP/6-311++G**[e]			53.0	
CCSD(T)/15s12p4d3f2g[f]			53.4	

[a]Basis sets selected for fitting are in parenthesis; [b]All NMR calculations were performed using the CCSD(T)/aug-pc-4 geometry (1.49466464 Å); [c]From ref.12; [d]From ref.62; [e]From ref.63; [f]From ref.64

Table S5A. ^{31}P isotropic shieldings in H_3PO calculated at the B3LYP, HF-SCF, and CCSD(T) level in combination with the aug-cc-pV*X*Z, aug-cc-pCV*X*Z, and aug-pcSseg-*n* basis sets.[a]

H$_3$PO				
**Basis set**	**b. f.**	**B3LYP**	**HF-SCF**	**CCSD(T)**
**aV*X*Z**				
D	77	458.078	494.411	496.373
T	165	381.595	431.022	427.424
Q	302	390.328	441.573	433.032
5	498	350.188	399.961	-
6	763	349.475	398.916	-
**CBS(5-6)**		**349.201**	**398.514**	**~396[b]**
**aCV*X*Z**				
D	90	402.100	447.671	451.545
T	203	354.785	403.727	397.616
Q	381	348.954	399.412	389.335
5	638	348.483	399.028	-
**CBS(T-5)**		**346.109**	**397.276**	**~388[b]**
**apcSseg-*n***				
0	39	453.796	517.119	510.616
1	86	377.240	424.750	423.473
2	180	354.333	403.097	393.977
3	345	348.303	398.614	405.597
4	563	348.178	398.391	-
**CBS(2-4)**		**347.681**	**396.549**	**~400[b]**

[a]B3LYP/aug-cc-pV5Z geometry of $H_3P=O$ was used (PO=1.476205; PH=1.412422 and HPO=116.746141); [b]A rough estimate from comparison of convergence patterns obtained with HF-SCF

Table S5B. The B3LYP, HF-SCF, and CCSD(T) energies (in a.u.) of H_3PO.

**H_3PO**				
**Basis set**	**b. f.**	**B3LYP**	**HF-SCF**	**CCSD(T)**
**aV*X*Z**				
D	77	-418.387596198	-417.338510362	-417.71138712
T	165	-418.442930694	-417.392246562	-417.84168225
Q	302	-418.457842226	-417.406265295	-417.88070359
5	498	-418.467265810	-417.412337212	-417.89555662
6	763	-418.468751518	-417.413440044	-417.90016792
**CBS(Q-6)**		**-418.46966928**	**-417.41397975**	**-417.9008092**
**aCV*X*Z**				
D	90	-418.394718919	-417.342068132	-417.71850259
T	203	-418.456313671	-417.401126555	-417.85452001
Q	381	-418.467766367	-417.411848696	-417.88841014
5	638	-418.469661666	-417.413678259	-417.89784511
**CBS(T-5)**		**-418.46997783**	**-417.41395163**	**-417.89721138**
**apcSseg*n***				
**1**	86	-418.376478710	-417.311315008	-417.68361574
**2**	180	-418.457227447	-417.398157629	-417.84214735
**3**	345	-418.469109581	-417.411219687	-417.88403616
**4**	563	-418.469750122	-417.412294093	-417.89429699
**CBS(2-4)**		**-418.47056645**	**-417.41303735**	**-417.893.84342**

Supplemental Material

Table S6A. Theoretical ^{23}Na nuclear shielding values (in ppm) of NaF and NaH calculated using various methods.

NaF			NaH			
**Basis set**	**b. f.**	**B3LYP**	**b. f.**	**B3LYP**	**HF-SCF**	**CCSD(T)**
**aV*X*Z**						
D	50	592.819	36	593.102	580.656	585.782
T	96	581.969	73	573.960	575.525	579.222
Q	164	579.192	130	574.010	563.720	565.539
5	258	572.578	211	559.698	563.753	541.040
**CBS(T-5)**		**574.674**		**565.305**	**562.384**	**549.057**
**aCV*X*Z**						
D	63	586.976	45	583.678	570.165	579.072
T	134	580.693	98	572.513	564.175	569.292
Q	243	580.332	180	572.662	565.081	569.674
5	398	580.362	297	572.697	565.24	569.408
**CBS(T-5)**		**580.311**		**572.698**	**565.269**	**569.555**
**apcSseg-*n***						
0	27	588.892	17	577.289	571.090	579.412
1	46	578.504	29	572.142	563.866	572.481
2	89	580.728	60	572.635	564.406	572.457
3	160	580.113	112	572.729	565.084	572.016
4	249	580.066	186	572.753	565.211	572.371
**CBS(2-4)**		**579.815**		**572.789**	**565.478**	**572.180**

Table S6B. Calculated energies (in a.u.) of NaF and NaH.

NaF		NaH	
**Basis set**	**B3LYP**	**B3LYP**	**CCSD(T)**
**aVXZ**			
D	-262.204869156	-162.86387918	-162.422001078
T	-262.237660615	-162.87141560	-162.44338745
Q	-262.247642152	-162.87414279	-162.45537297
5	-262.251590656	-162.87510466	-162.45892442
**CBS(T-5)**	**-262.25527211**	**-162.87612472**	**-162.46348953**
**aCVXZ**			
D	-262.20827680	-162.86540524	-162.61455950
T	-262.24443261	-162.87621861	-162.71919341
Q	-262.25322055	-162.87802768	-162.77681296
5	-262.25542162	-162.87831464	-162.80221667
**CBS(T-5)**	**-262.25881776**	**-162.87903395**	**-162.82315118**

Table S7A. ^{25}Mg nuclear shielding values (in ppm) of MgH_2 calculated at the HF-SCF, B3LYP, and CCSD(T) level, combined with the aug-cc-pV*X*Z, aug-cc-pCV*X*Z , aug-pcSseg-*n*, and x2c-Def2 basis sets.

MgH_2				
**Basis set**	**b. f.**	**B3LYP**	**HF-SCF**	**CCSD(T)**
**aV*X*Z**				
D	45	486.035	503.602	496.843
T	96	459.192	488.420	473.084
Q	176	421.519	485.928	469.522
5	291	388.385	468.158	423.764
**CBS(T-5)**		**397.813**	**475.048**	**441.802**
**aCV*X*Z**				
D	54	473.001	489.815	484.616
T	121	439.339	470.668	457.479
Q	226	432.012	465.987	451.201
5	377	423.201	461.617	445.393
**CBS(T-5)**		**426.089**	**462.948**	**447.156**
**apcSseg-*n***				
0	20	459.402	486.743	467.466
1	38	426.175	462.991	450.642
2	83	426.234	459.800	447.809
3	161	426.427	460.467	444.129
4	272	426.676	460.780	444.226
**CBS(2-4)**		**426.588**	**460.706**	**443.870**
**x2c-*X*ZVPPall-s**				
x2c-SVPall-s	37	425.167	462.634	450.466
x2c-TZVPPall-s	69	426.580	460.266	449.526
x2c-QZVPPall-s	135	423.667	457.168	443.334
**CBS(1-3)**		**424.985**	**458.225**	**445.784**

Table S7B. The B3LYP and CCSD(T) energies (in a.u.) of MgH_2 calculated with the aug-cc-pV*X*Z and aug-cc-pCV*X*Z basis set series.

**MgH_2**		
**Basis set**	**B3LYP**	**CCSD(T)**
**aV*X*Z**		
D	-201.25953842	-200.80399245
T	-201.26997915	-200.84155803
Q	-201.27228212	-200.85863579
5	-201.27465395	-200.92482476
**CBS(T-5)**	**-201.27532589**	**-200.92390375**
**aCV*X*Z**		
D	-201.2615830	-200.975948470
T	-201.2731550	-201.120087868
Q	-201.2757430	-201.17385263
5	-201.2766084	-201.19787045
**CBS(T-5)**	**-201.2775821**	**-201.21736633**

Table S8A. ^{27}Al nuclear shielding values (in ppm) of AlH_3 calculated at the HF-SCF, B3LYP, and CCSD(T) level, combined with the aug-cc-pV*X*Z, aug-cc-pCV*X*Z , aug-pcSseg-*n*, and x2c-Def2 basis sets..

**AlH_3**				
**Basis set**	**b. f.**	**B3LYP**	**HF-SCF**	**CCSD(T)**
**aV*X*Z**				
D	54	356.842	418.019	398.363
T	119	267.533	352.723	324.075
Q	222	279.900	365.604	319.626
5	371	264.302	344.518	305.039
6	574	261.701	342.906	Not conv.
**CBS(Q-6)**		**260.370**	**340.417**	**301.061**
**aCV*X*Z**			**awCV*X*Z**	**awCV*X*Z**
D	63	292.346	350.971	318.748
T	144	270.502	348.433	311.521
Q	272	268.116	347.181	308.498
5	457	266.955	346.512	307.726
**CBS(T-5)**		**267.211**	**346.671**	**307.762**
**apcSseg-*n***				
0	26	224.919	321.006	264.706
1	60	272.72	347.879	312.927
2	128	266.47	344.749	307.232
3	172	264.03	341.332	305.189
4	414	266.649	346.004	306.424
**CBS(2-4)**		**265.630**	**344.362**	**305.775**
**x2c-*X*ZVPPall-s**				
x2c-SVPall-s	45	255.610	338.958	306.507
x2c-TZVPPall-s	96	268.910	346.915	311.762
x2c-QZVPPall-s	172	261.219	341.332	305.189
**CBS(1-3)**		**265.443**	**344.293**	**308.421**

Table S8B. The B3LYP and CCSD(T) energies (in a.u.) of AlH_3 calculated with the aug-cc-pV*X*Z and aug-cc-pCV*X*Z basis set series.

**AlH_3**		
**Basis set**	**B3LYP**	**CCSD(T)**
**aV*X*Z**		
D	-244.21922425	-243.747570184
T	-244.23360255	-243.837834800
Q	-244.23939987	-243.83729289
5	-244.24097337	-243.92943312
6	-244.24104531	-
**CBS(Q-6)**	**-244.24197328**	**-**
**CBS(T-5)**	**-**	**-243.91801437**
**aCV*X*Z**		
D	-244.22201311	-243.87445191
T	-244.23785921	-244.04698171
Q	-244.24170076	-244.12311882
5	-244.24227256	-244.15894233
**CBS(T-5)**	**-244.24380456**	**-244.18633069**

Table S9A. ^{29}Si nuclear shielding values (in ppm) of SiH_4 calculated at the HF-SCF, B3LYP, and CCSD(T) level, combined with the aug-cc-pV*X*Z, aug-cc-pCV*X*Z , aug-pcSseg-*n*, and x2c-Def2 basis sets..

**SiH_4**				
**Basis set**	**b. f.**	**B3LYP**	**HF-SCF**	**CCSD(T)**
**aV*X*Z**				
D	63	524.779	548.033	554.575
T	142	450.243	485.124	488.826
Q	268	457.562	506.361	501.517
5	451	435.668	473.932	468.513
**CBS(T-5)**		**445.370**	**489.275**	**483.294**
**aCV*X*Z**				
D	72	473.6513	507.218	516.049
T	167	439.0493	476.680	476.451
Q	318	436.0286	474.938	471.664
5	537	435.4506	479.932	-
**CBS(T-5)**		**435.416**	**477.703**	**470.854**
**apcSseg-*n***				
0	29	486.947	533.581	540.468
1	69	444.030	483.088	483.900
2	151	436.940	475.284	469.321
3	297	434.980	473.826	469.018
4	500	435.270	473.974	-
**CBS(2-4)**		**434.990**	**473.79**	**468.972**
**x2c-*X*ZVPPall-s**				
x2c-SVPall-s	53	441.080	480.403	482.139
x2c-TZVPPall-s	113	437.239	475.830	472.508
x2c-QZVPPall-s	205	431.572	470.8032	467.086
**CBS(1-3)**		**433.841**	**472.744**	**468.890**

Table S9B. The B3LYP and CCSD(T) energies (in a.u.) of SiH_4

**SiH_4**		
**Basis set**	**B3LYP**	**CCSD(T)**
**aV*X*Z**		
D	-291.89879366	-291.4087569985
T	-291.91847211	-291.5056560050
Q	-291.92448059	-291.504971737
5	-291.92810244	-291.627661525
6	-291.92866588	-
**CBS(Q-6)**	**-291.93081935**	**-**
**CBS(T-5)**		**-291.61247239**
**aCV*X*Z**		
D	-291.90205678	-291.607682461
T	-291.92365987	-291.765560806
Q	-291.92870585	-291.827830430
5	-291.92937201	-
**CBS(T-5)**	**-291.93139466**	**-**
**CBS(D-Q)**	**-**	**-291.87327043**

Table S9C. ^{29}Si nuclear shielding values (in ppm) of HSiCH calculated at the HF-SCF, B3LYP, and CCSD(T) level, combined with the aug-cc-pV*X*Z, aug-cc-pCV*X*Z , and aug-pcSseg-*n* basis sets..

**HSiCH**				
**Basis set**	**b. f.**	**B3LYP**	**HF-SCF**	**CCSD(T)**
**aV*X*Z**				
D	68	556.6846	905.475	666.826
T	142	505.9667	903.962	634.782
Q	256	511.876	905.158	641.418
5	418	499.5118	914.777	624.410

6	636	501.1438	-	-
**CBS(T-5,6)**		**498.605**	**917.646**	**619.338**
**aCV*X*Z**				
D	81	514.935	900.384	645.931
T	180	502.124	904.667	630.532
Q	335	501.597	907.255	630.168
5	558	501.171	907.519	-
**CBS(T-5)**		**501.293**	**907.666**	**630.101**
**apcSseg*n***				
1	77	503.7813	922.885	644.709
2	157	500.7014	914.772	625.185
3	296	501.4064	916.405	628.228
4	477	501.7846	914.661	-
**CBS(2-4)**		**501.697**	**915.536**	**628.762**

Table S10A. ^{33}S nuclear shielding values (in ppm) of H_2S calculated at the HF-SCF, B3LYP, and CCSD(T) level, combined with the aug-cc-pV*X*Z, aug-cc-pCV*X*Z , aug-pcSseg-*n*, and x2c-Def2 basis sets..

**H_2S**				
**Basis set**	**b. f.**	**B3LYP**	**HF-SCF**	**CCSD(T)**
**aV*X*Z**				
D	45	757.263	768.299	794.741
T	96	721.620	738.199	765.513
Q	176	722.836	736.350	762.929
5	291	699.124	712.597	740.623
6	447	698.200	712.258	740.020
**CBS(Q-6)**		**694.933**	**708.776**	**736.852**
**aCV*X*Z**				
D	54	726.299	740.258	770.194
T	121	702.600	716.827	745.570
Q	226	698.785	713.141	741.832
5	377	698.501	712.906	741.393
**CBS(T-5)**		**698.246**	**712.644**	**741.209**
**apcSseg-*n***				
0	23	746.908	793.434	804.953
1	51	709.21	726.433	755.754
2	105	700.97	715.123	739.502
3	199	698.26	719.781	743.898
4	328	698.37	712.476	740.381
**CBS(2-4)**		**698.071**	**715.929**	**742.245**
**x2c-*X*ZVPPall-s**				
x2c-SVPall-s	37	728.757	755.384	770.757
x2c-TZVPPall-s	79	701.935	719.781	743.898
x2c-QZVPPall-s	139	694.072	709.531	736.967
**CBS(1-3)**		**695.877**	**711.844**	**738.358**

Table S10B. The B3LYP and CCSD(T) energies (in a.u.) of H_2S.

**H_2S**		
**Basis set**	**B3LYP**	**CCSD(T)**
**aV*X*Z**		
D	-399.41468530	-398.88839956
T	-399.43217564	-398.97922094
Q	-399.43649626	-399.01740712
5	-399.44059810	-399.13954634
6	-399.44111883	-399.21382420
**CBS(Q-6)**	**-399.44355253**	**-399.28887450**
**aCV*X*Z**		
D	-399.41755048	-399.09628617
T	-399.43695573	-399.27659357
Q	-399.44115027	-399.34894772
5	-399.44175495	-399.37832694
**CBS(T-5)**	**-399.44343012**	**-399.40492109**

Table S10C. The B3LYP calculated ^{33}S nuclear shieldings for 2-thiouracil with aug-cc-pVXZ and aug-cc-pCVXZ basis sets on all atoms and within LDBS approach (all atoms with 6-31G* basis set and only sulfur with aVXZ and aCVXZ)

**2-TU**								
**Basis set**	**All**				**LDBS**			
(X)		H, C, O, N, S: aVXZ		H: aVXZ; C, O, N, S: aCVXZ	(C, O, N and H: 6-31G*)			
	bf	B3LYP/aVXZ	bf		bf	S: aVXZ	bf	S: aCVXZ
D	224	387.869	261	331.045	133	398.795	142	347.543
T	464	335.860	580	306.502	156	365.034	181	332.193

Q	828	343.732	1081	301.800	190	371.328	240	327.708
5	1340	299.099	1804	301.169	237	325.067	323	327.367
6	2024	298.830	-	-	299	325.205	-	-
**CBS**		**298.720**		**300.970**		**325.342**		**325.774**
**6-31G***	**132**	**378.514**						

Table S11A. ^{35}Cl nuclear shielding values (in ppm) of HCl calculated at the HF-SCF, B3LYP, and CCSD(T) level, combined with the aug-cc-pV*X*Z, aug-cc-pCV*X*Z , aug-pcSseg-*n*, and x2c-Def2 basis sets..

**HCl**				
**Basis set**	**b. f.**	**B3LYP**	**HF-SCF**	**CCSD(T)**
**aV*X*Z**				
D	36	961.8332	977.258	986.763
T	73	945.2023	961.547	970.798
Q	130	944.7120	958.593	968.566
5	211	932.9930	946.919	957.914
6	320	931.7249	946.089	957.249
**CBS(Q-6)**		**930.256**	**944.476**	**955.745**
**aCV*X*Z**				
D	45	945.4964	961.169	972.344
T	98	933.9553	948.667	958.910
Q	180	932.1409	946.678	958.007
5	297	931.9804	946.561	958.055
**CBS(Q-6)**		**931.858**	**946.403**	**957.943**
**apcSseg-*n***				
0	20	941.602	980.478	977.953
1	42	935.83	953.008	964.124
2	82	934.08	948.579	956.537
3	150	931.96	946.336	957.071
4	242	931.91	946.272	957.442
**CBS(2-4)**		**931.705**	**946.060**	**957.3498**
**x2c-*X*ZVPPall-s**				
x2c-SVPall-s	29	982.965	982.965	983.097
x2c-TZVPPall-s	62	957.132	957.131	963.485
x2c-QZVPPall-s	106	944.420	944.419	954.303

**CBS(1-3)**		**948.413**	**948.412**	**957.127**

Table S11B. The B3LYP and CCSD(T) energies (in a.u.) of HCl

**HCl**		
**Basis set**	**B3LYP**	**CCSD(T)**
**aV*X*Z**		
D	-460.82758530	-460.27881668
T	-460.84426235	-460.37795747
Q	-460.84818174	-460.42666503
5	-460.85164037	-460.54386650
6	-460.85220096	-460.62888464
**CBS(Q-6)**	**-460.85425837**	**-460.70150484**
**aCV*X*Z**		
D	-460.830248867	-460.490880368
T	-460.848259238	-460.68548124
Q	-460.852219393	-460.76287445
5	-460.852812828	-460.79423489
**CBS(T-5)**	**-460.85439165**	**-460.82268904**

Table S12A. ^{39}Ar nuclear magnetic shielding values (in ppm) calculated for an isolated atom at the HF-SCF, B3LYP, and CCSD(T) level, combined with the aug-cc-pV*X*Z, aug-cc-pCV*X*Z , aug-pcSseg-*n*, and x2c-Def2 basis sets..

**Ar**				
**Basis set**	**b. f.**	**B3LYP**	**HF-SCF**	**CCSD(T)**
**aV*X*Z**				
D	27	1237.382	1237.602	1237.071
T	50	1237.057	1237.615	1237.246
Q	84	1237.121	1237.651	1237.258
5	131	1237.944	1237.657	1237.394
6	193	1238.049	1237.658	1237.524
**CBS(Q-6)**		**1238.172**	**1237.659**	**1237.509**
**aCV*X*Z**				
D	36	1237.399	1237.570	1237.119
T	75	1237.610	1237.621	1237.644
Q	134	1237.836	1237.655	1237.846
5	217	1237.846	1237.657	1237.937
**CBS(T-5)**		**1237.868**	**1237.660**	**1237.924**
**apcSseg-*n***				
0	17	1236.404	1236.578	1236.178
1	33	1237.716	1237.155	1236.677
2	59	1237.881	1237.269	1237.004
3	101	1237.936	1237.507	1237.404
4	156	1237.915	1237.498	1237.497
**CBS(2-4)**		**1237.930**	**1237.534**	**1237.516**
**x2c-*X*ZVPPall-s**				
x2c-SVPall-s	21	1249.189	1248.648	1248.437
x2c-TZVPPall-s	45	1249.336	1248.803	1248.634
x2c-QZVPPall-s	73	1236.887	1236.677	1236.579
**CBS(1-3)**		**1242.409**	**1242.056**	**1241.920**

Table S12B. The B3LYP and CCSD(T) energies (in a.u.) of isolated Ar atom**.**

Ar		
**Basis set**	**B3LYP**	**CCSD(T)**
**aV*X*Z**		
D	-527.54538899	-526.97518862
T	-527.56000196	-527.08028270
Q	-527.56367703	-527.13763465
5	-527.56633502	-527.25062305
6	-527.56703390	-527.34980274
**CBS(Q-6)**	**-527.56805707**	**-527.38485741**
**aCV*X*Z**		
D	-527.54773771	-527.19113752
T	-527.56317741	-527.39744948
Q	-527.56703529	-527.48090156
5	-527.56763687	-527.51450674
**CBS(T-5)**	**-527.56917203**	**-527.54521404**

Table S13. Relativistic corrections of PN (in ppm) calculated at the B3LYP/aug-cc-pV*X*Z level (X = D, T, Q)

**B3LYP/aV*X*Z**	**PN**
D	29.82
T	19.50
Q	12.37

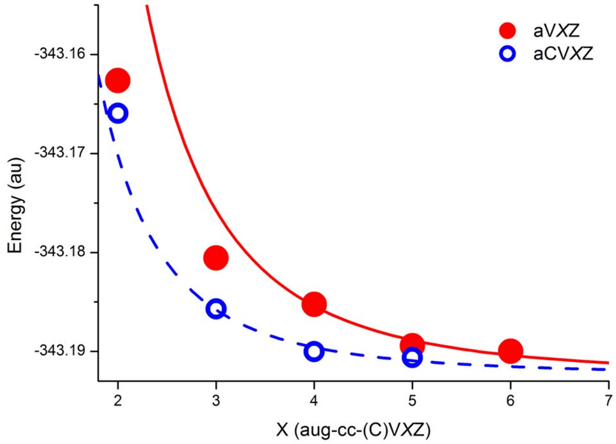

Figure S1. Convergences of the B3LYP/aug-cc-pV*X*Z and B3LYP/aug-cc-pCV*X*Z energies of PH_3 with fitting lines shown.

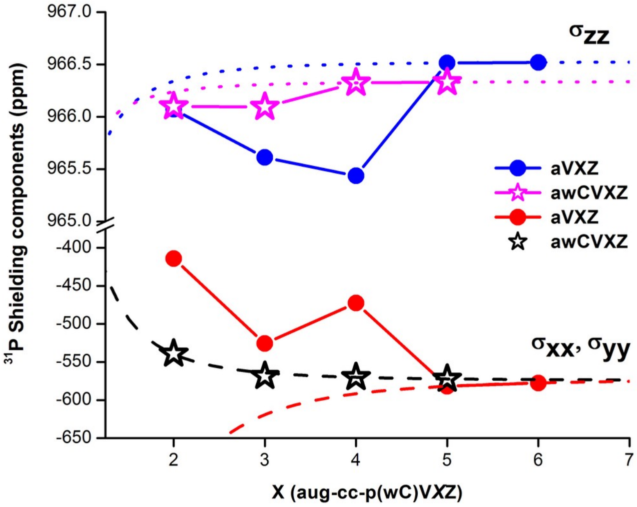

$\sigma_{xx} = \sigma_{yy}$

Figure S2. Theoretical σ_{xx} (= σ_{yy}) and σ_{zz} components of ^{31}P nuclear shielding constants in PN calculated at the B3LYP/aug-cc-pV*X*Z and the B3LYP/aug-cc-pwCV*X*Z levels, where *X*=2 – 5 or 6. The fitting curves estimated with the 2-parameter formula are shown as well.

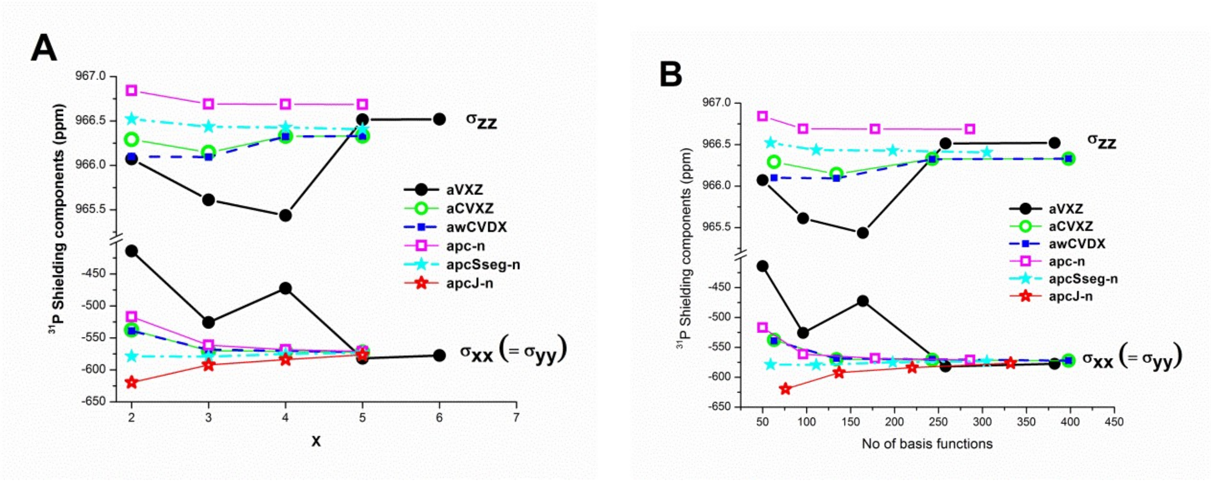

Figure S3. B3LYP calculated σ_{xx} (= σ_{yy}) and σ_{zz} components of the ^{31}P shielding constants of PN calculated with selected Dunning's and Jensen's basis set families plotted against (A) the cardinal number *X* and (B) the number of basis functions.

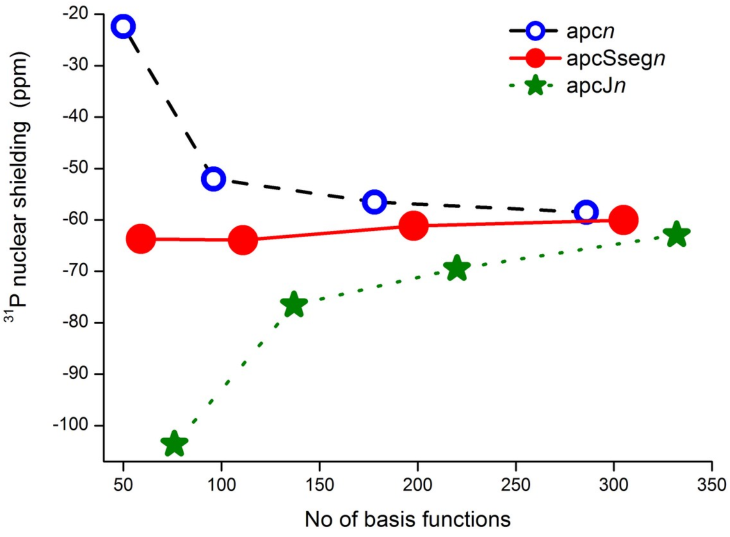

Figure S4. Performance of Jensen's basis sets using the B3LYP functional in prediction of ^{31}P shielding in PN.

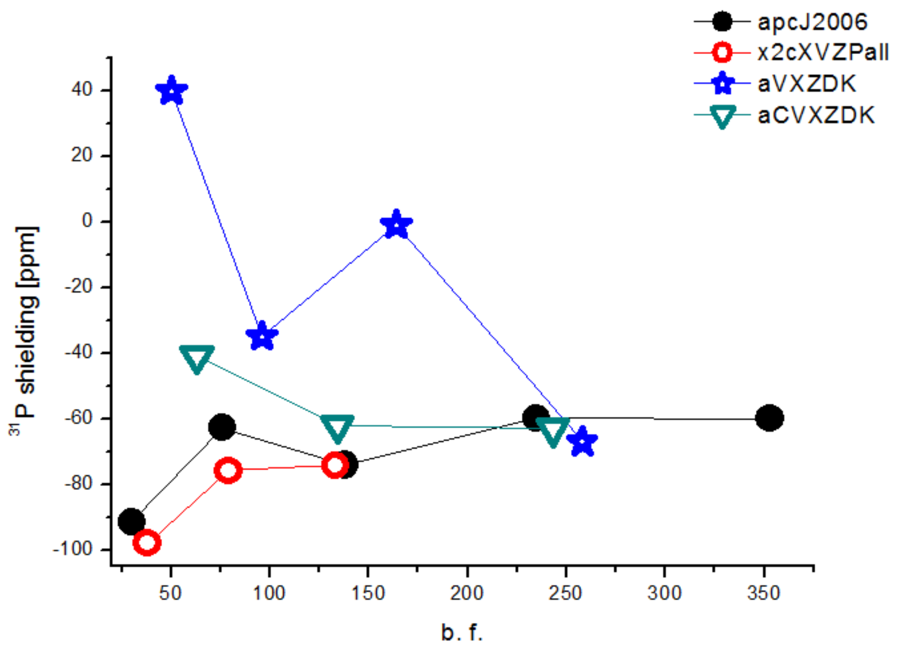
apcJ2006
x2cXVZPall
aVXZDK
aCVXZDK
40
20
0
-20
-40
-60
-80
-100
^{31}P shielding [ppm]
50
100
150
200
250
300
350
b. f.

Figure S5. Convergence of ^{31}P shieldings in PN calculated with the B3LYP functional and the aug-pcJ2006, x2c-Def2, aug-cc-pV*X*Z-DK, and aug-cc-pCV*X*Z-DK basis set series.

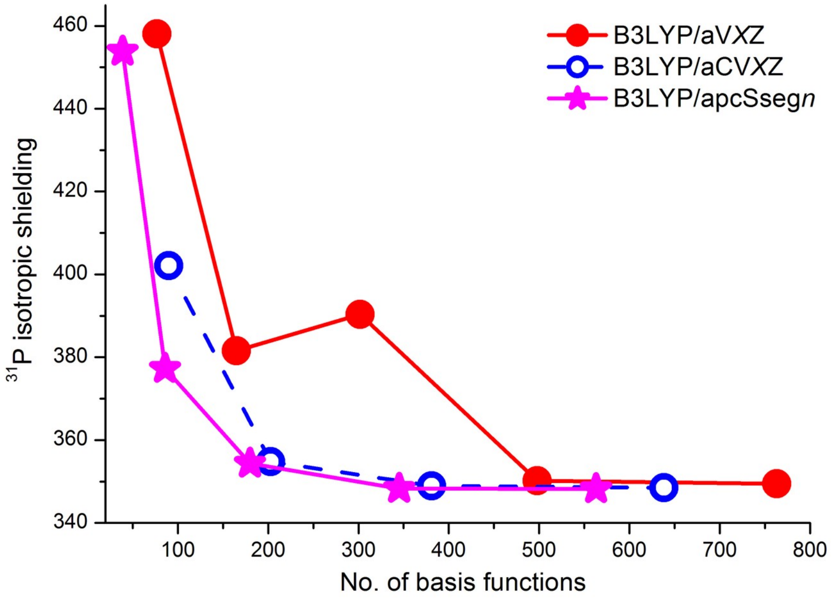

Figure S6. Convergence patterns of ^{31}P isotropic shieldings in H_3PO calculated with B3LYP and HF-SCF in combination with aug-cc-pV*X*Z basis set series and B3LYP/aug-pcSseg-*n* level of theory.

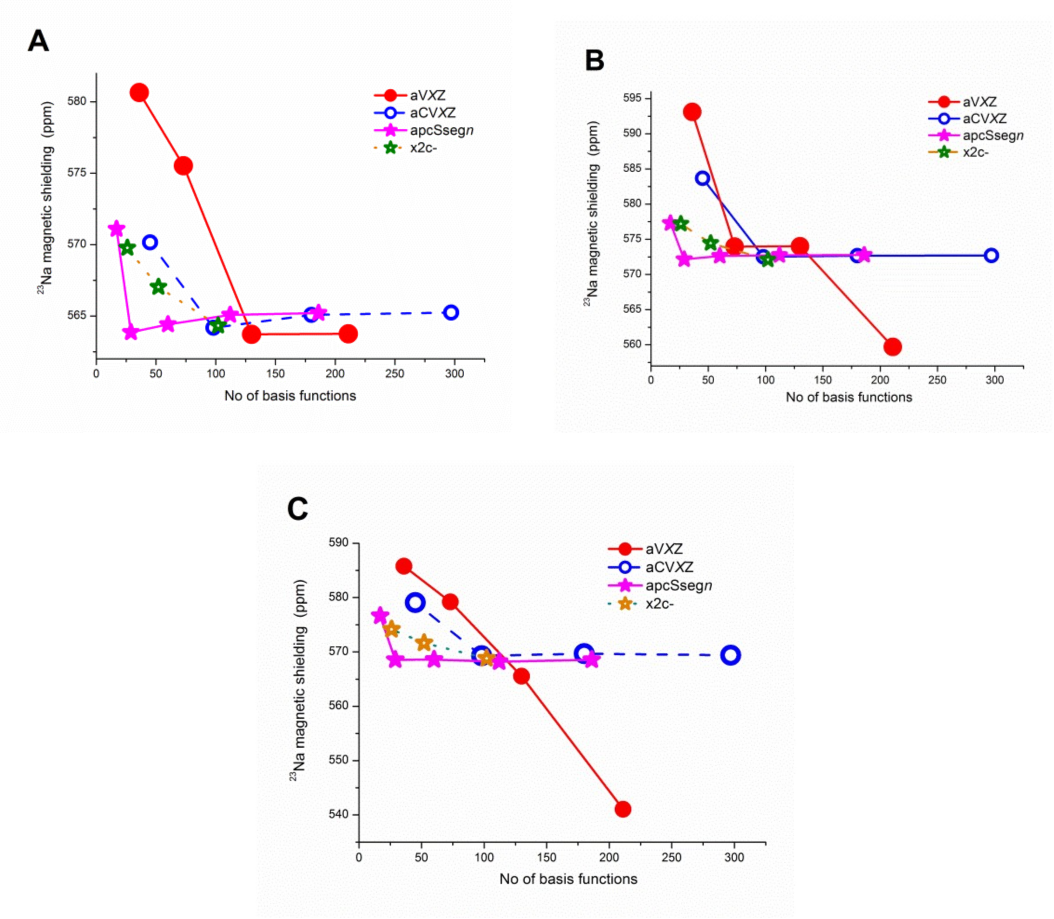

Figure S7. ^{23}Na isotropic shielding constants for NaH calculated with the (A) HF-SCF, (B) B3LYP and (C) CCSD(T) methods, using the aug-cc-pV*X*Z, aug-cc-pCV*X*Z, aug-pcSseg-*n* and x2c-Def2 basis set families.

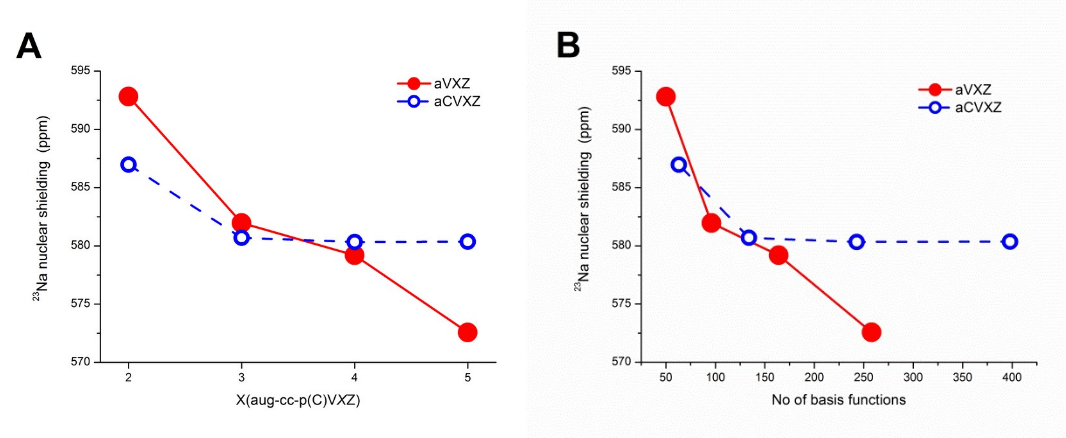

Figure S8. The B3LYP ^{23}Na shielding constants with respect to (A) the cardinal number X and (B) the number of basis functions for NaF calculated with the aug-cc-pV*X*Z and aug-cc-pCVXZ basis set families.

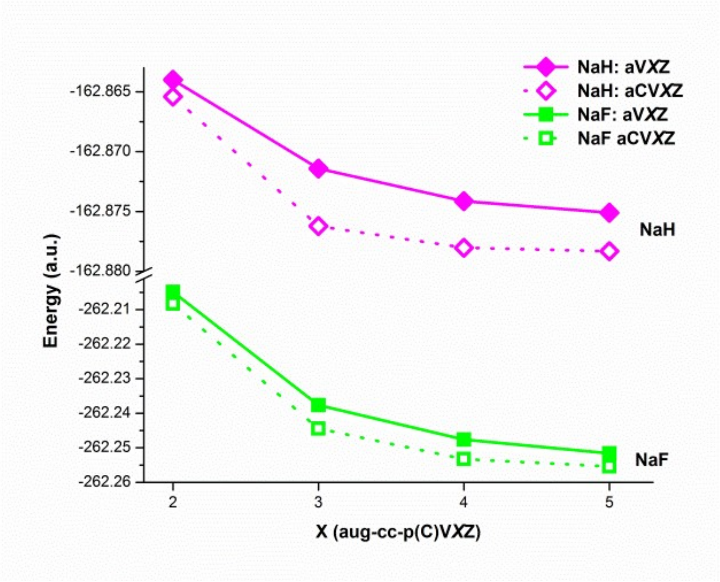

Figure S9. Convergence of the B3LYP/aug-cc-pV*X*Z and B3LYP/aug-cc-pCV*X*Z energies of NaH and NaF.

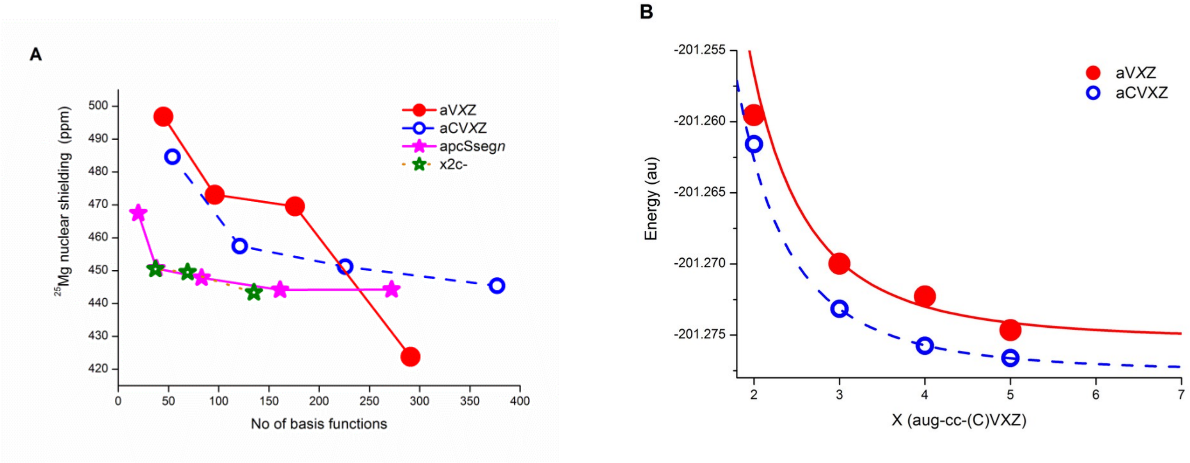

Figure S10. Convergence of ^{25}Mg isotropic shielding constants for MgH_2 calculated with the CCSD(T) method using the aug-cc-pV*X*Z, aug-cc-pCV*X*Z, aug-pcSseg-*n* and x2c-*X*ZVPall-s basis set families (A). Convergences of the B3LYP energies of MgH_2 calculated with the aug-cc-pV*X*Z and aug-cc-pCV*X*Z basis sets are on the right (B)

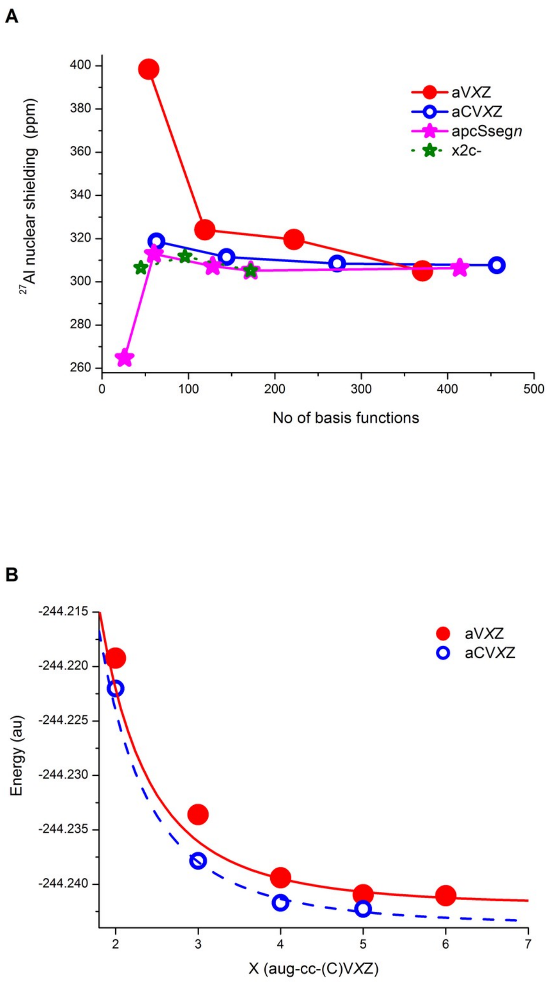

Figure S11. ^{27}Al isotropic shielding constants for AlH_3 predicted with the CCSD(T) method and the aug-cc-pV*X*Z, aug-cc-pCV*X*Z, aug-pcSseg-*n*, and x2c-*X*ZVPall-s basis set families (A). The B3LYP/aug-cc-pV*X*Z and B3LYP/aug-cc-pCV*X*Z calculated energies of AlH_3 are on the right (B).

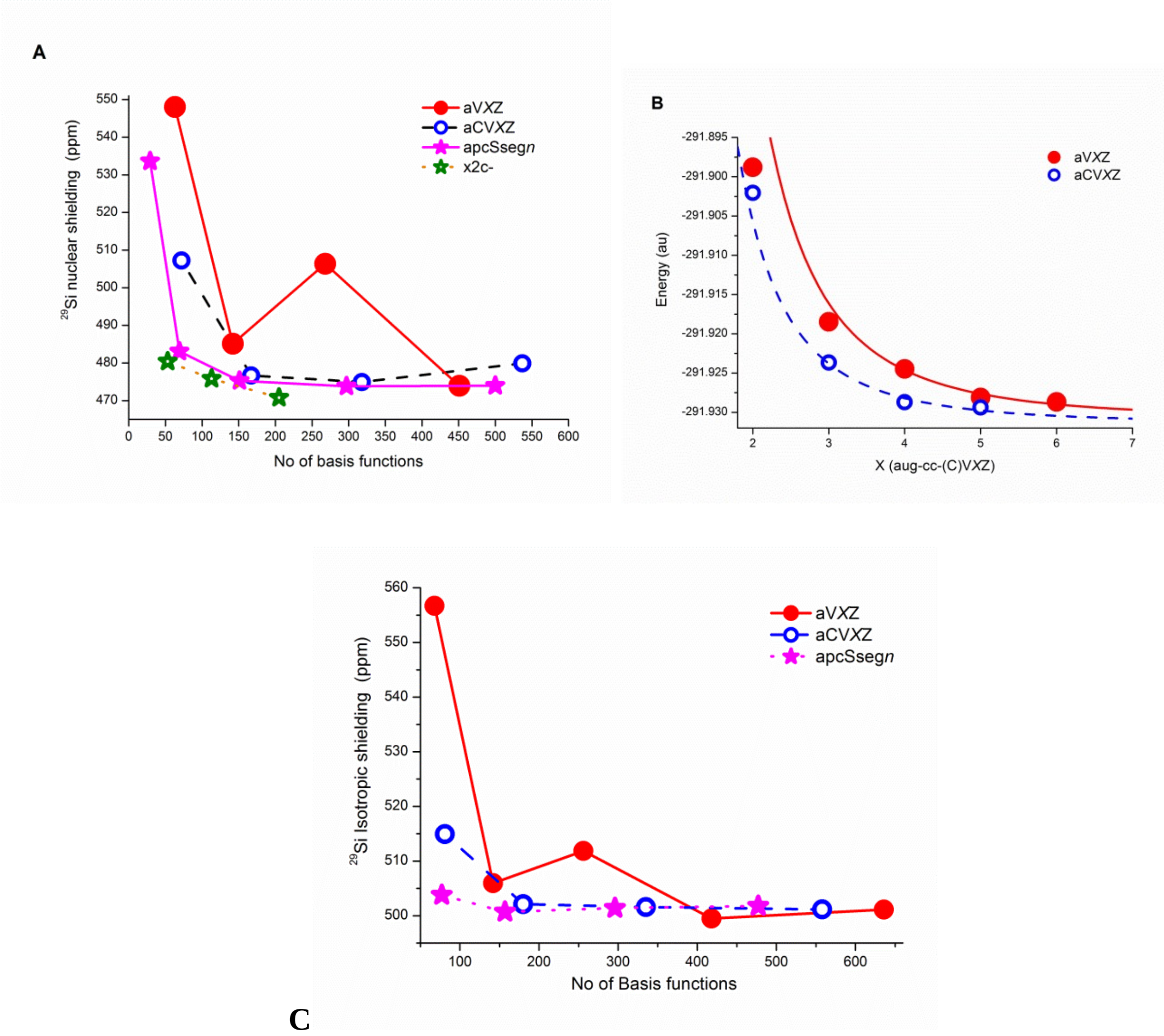

Figure S12. ^{29}Si isotropic shielding constants for SiH_4 calculated with the HF-SCF method and the aug-cc-pV*X*Z, aug-cc-pCV*X*Z, aug-pcSseg-*n*, and x2c-*X*ZVPall-s basis set families (A). Convergences of the B3LYP/aug-cc-pV*X*Z and B3LYP/aug-cc-pCV*X*Z energies of SiH_4 are on the right (B) and ^{29}Si isotropic shielding constants for HSiCH, calculated with the B3LYP density functional combined with the aug-cc-pV*X*Z and aug-pcSseg-*n*, basis set families (C).

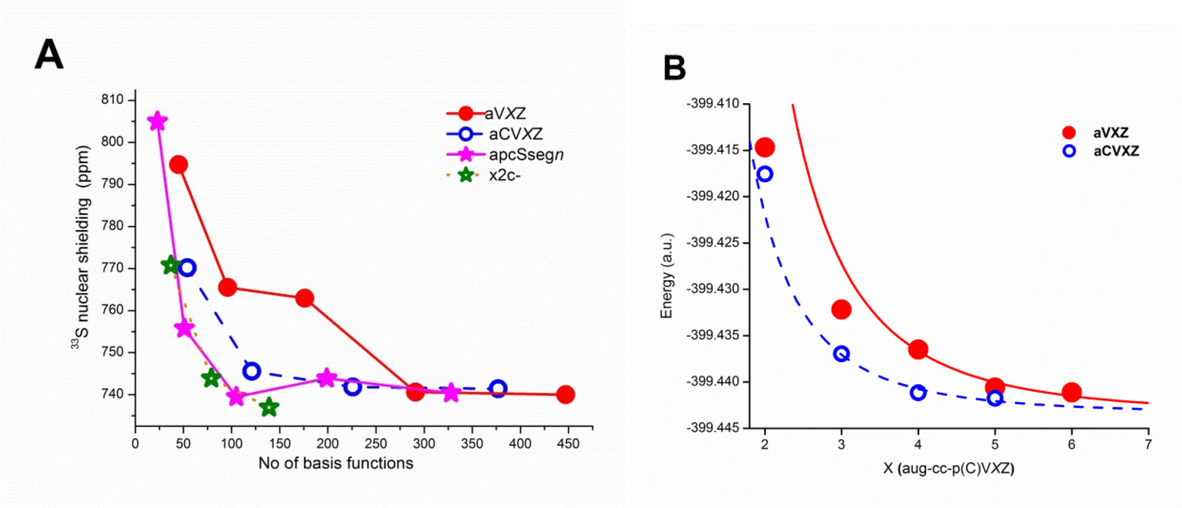

Figure S13. ^{33}S isotropic shielding constants for H_2S calculated with the CCSD(T) method and the aug-cc-pV*X*Z, aug-cc-pCV*X*Z, aug-pcSseg-*n*, and x2c-*X*ZVPall-s basis set families (A). Convergences of the B3LYP/aug-cc-pV*X*Z and B3LYP/aug-cc-pCV*X*Z energies of H_2S are on the right (B).

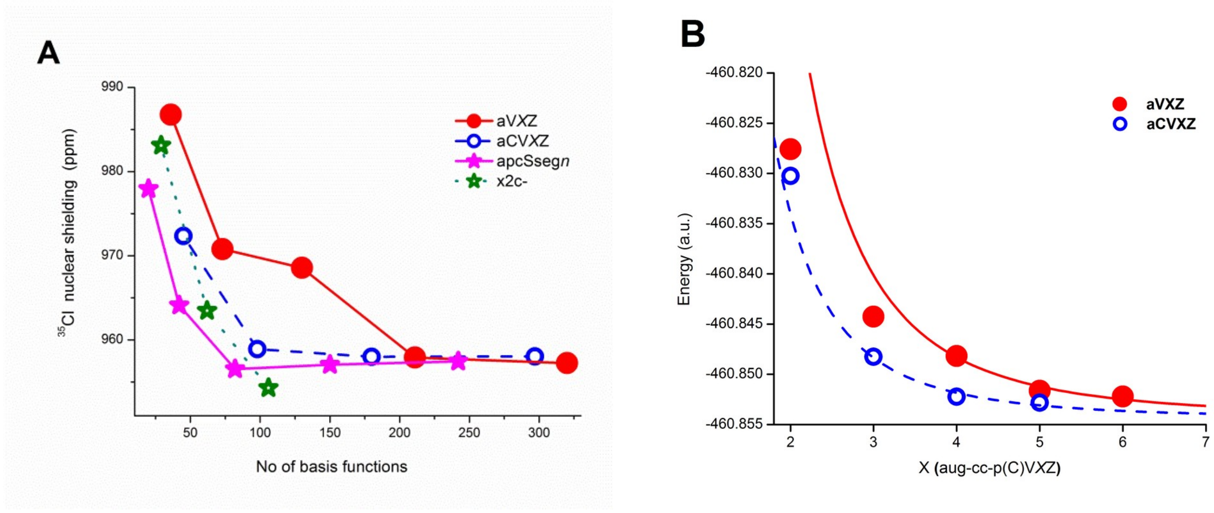

Figure S14. ^{35}Cl isotropic shielding constants for HCl calculated with the CCSD(T) method and the aug-cc-pV*X*Z, aug-cc-pCV*X*Z, aug-pcSseg-*n*, and x2c-*X*ZVPall-s basis set families (A). Convergences of the B3LYP/aug-cc-pV*X*Z and B3LYP/aug-cc-pCV*X*Z energies of HCl are on the right (B).

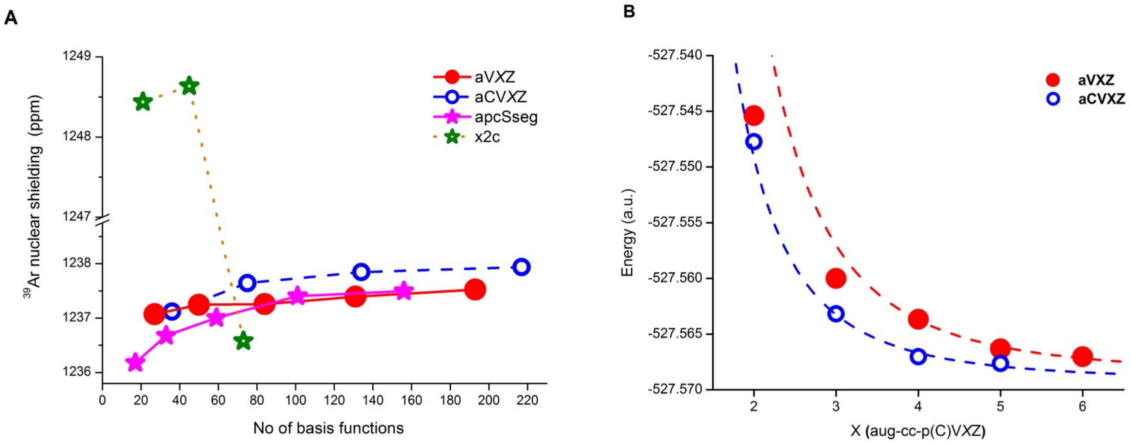

Figure S15. ^{39}Ar isotropic shielding constants for isolated argon atom calculated with the CCSD(T) method using the aug-cc-pV*X*Z, aug-cc-pCV*X*Z, aug-pcSseg-*n*, and x2c-Def2 basis set families (A). Convergences of the B3LYP/aug-cc-pV*X*Z and B3LYP/aug-cc-pCV*X*Z energies of Ar are on the right (B).
